# Distinct subcortical neuroanatomic profiles of treatment-resistant schizophrenia: structural magnetic resonance imaging study

**DOI:** 10.1192/bjo.2025.10939

**Published:** 2026-01-12

**Authors:** Ibrahim Sungur, Simay Selek, Kaan Keskin, Asli Ceren Hinc, Furkan Yazici, Elif Ozge Aktas, Yigit Erdogan, Omer Kitis, Ali Saffet Gonul

**Affiliations:** SoCAT Lab, Department of Psychiatry, School of Medicine, Ege University, Izmir, Turkey; Department of Psychiatry, Ege University, Izmir, Turkey; Department of Psychiatry, Izmir City Hospital, Izmir, Turkey; Department of Radiology, Ege University, Izmir, Turkey; School of Medicine, Department of Psychiatry and Behavioral Sciences, Mercer University, Mocon, Georgia, USA

**Keywords:** Neuroimaging, MRI, schizophrenia, clozapine, hippocampus

## Abstract

**Background:**

Understanding the neuroanatomical correlates of treatment response in schizophrenia is crucial for improving clinical stratification and clarifying underlying pathophysiological mechanisms.

**Aims:**

To examine subcortical volumetric differences across clinically defined schizophrenia treatment-response subgroups.

**Method:**

T1-weighted structural magnetic resonance imaging data were analysed from 109 participants, including 79 individuals with schizophrenia and 30 healthy controls. Patients were categorised into three distinct treatment response groups: ultra-treatment-resistant (UTR; *n* = 22), clozapine-responsive (*n* = 28) and first-line antipsychotic responsive (FLR; *n* = 29). Group differences were examined across 33 regions of interest, including subcortical, ventricular and hippocampal subfield regions.

**Results:**

The UTR group had higher antipsychotic dosages and exhibited greater symptom severity than other patient groups. Across all schizophrenia subgroups, hippocampal and amygdala volumes were smaller relative to controls. Treatment-resistant patients (UTR and clozapine-responsive) also showed reduced nucleus accumbens volumes, whereas FLR patients demonstrated larger pallidal volumes. In addition, the UTR subgroup exhibited enlarged lateral ventricles. Hippocampal subfield analyses revealed widespread reductions in treatment-resistant patients, most prominently in the CA4/dentate gyrus, subiculum and stratum, whereas FLR patients showed more focal reductions in the CA4/dentate gyrus and left subiculum.

**Conclusions:**

These results suggest that smaller hippocampal and amygdala volumes represent a shared neuroanatomical signature of schizophrenia, whereas reduced accumbens and enlarged pallidal volumes may differentiate treatment-resistant and treatment-responsive profiles, respectively. The findings underscore the heterogeneity of schizophrenia and highlight the need for longitudinal research to disentangle illness-related pathology from medication effects.

Schizophrenia is a heterogeneous psychiatric disorder characterised by considerable variability in symptom profiles, illness trajectories and treatment outcomes.^1^ Despite advances in antipsychotic treatments, approximately a third of patients with schizophrenia fail to achieve adequate symptom remission and are classified as having treatment-resistant schizophrenia (TRS).^2^ Treatment resistance is typically defined by persistent symptoms despite adequate trials of at least two different antipsychotic medications at sufficient doses and durations. Clozapine remains the gold standard medication for TRS because of its unique mechanism of action. However, up to 40–60% of the treatment-resistant patients do not respond adequately even to clozapine, representing a subgroup referred to as ultra-treatment-resistant (UTR).^3^ This high rate of clozapine non-response underscores the existence of further heterogeneity within TRS. Most prior work has employed a binary classification of TRS versus non-TRS, potentially overlooking clinically meaningful heterogeneity among treatment response subtypes. This approach limits the ability to identify neurobiological differences among groups such as first-line antipsychotic responsive (FLR), clozapine-responsive and UTR patients.^4^ More refined stratification may yield deeper insights into the biological underpinnings of treatment response variability and disease heterogeneity.

Structural magnetic resonance imaging (MRI) studies have consistently reported widespread grey matter reductions in schizophrenia, particularly affecting frontotemporal and subcortical regions such as the hippocampus, amygdala and thalamus.^5,6^ However, these findings have been highly heterogeneous, potentially because of differences in sample characteristics, illness duration, treatment exposure and inconsistent definitions of treatment resistance.^7^ Emerging evidence suggests that patients with TRS may show more pronounced and regionally specific structural abnormalities, particularly within limbic and subcortical circuits implicated in emotional regulation and cognitive control.^4,7,8^ Furthermore, hippocampal volume loss is among the most consistently reported structural alterations in schizophrenia.^9,10^ Notably, a recent pseudo-longitudinal study described a hippocampal atrophy subtype characterised by early volume loss in the hippocampus, with subsequent extension to other limbic regions such as the amygdala and striatum.^11^ Previous studies also indicate that these alterations are detectable not only at the whole-structure level, but also within specific hippocampal subfields.^10,12^

In this study, we aimed to examine structural brain differences among patients with schizophrenia categorised into UTR, clozapine-responsive and FLR subgroups, compared with healthy controls. We hypothesised that patients with treatment-resistant profiles (UTR and clozapine-responsive) would exhibit more pronounced subcortical volumetric abnormalities than FLR patients, with the greatest reductions expected in the limbic regions and basal ganglia, particularly in the hippocampus and its subfields, relative to healthy controls.^8,10,13^ Although subfield-level analyses can be extended to other regions, evidence linking hippocampal subfields to treatment resistance is considerably more consistent than that for thalamic or amygdala nuclei.^8,10,14^ Furthermore, the hippocampus plays a pivotal role in fronto-hippocampal circuits regulating mesolimbic dopamine transmission, and its dysfunction has been proposed as a core mechanism driving dopaminergic dysregulation in schizophrenia.^15^

## Method

### Participants

This study included 79 patients with schizophrenia (mean age±s.d.: 37.5±9.9) and 30 right-handed healthy controls (mean age±s.d.: 39.3±8.9). Patients were recruited from the out-patient and in-patient units of the Department of Psychiatry, Ege University School of Medicine, Izmir, Turkey. Written informed consent was obtained from patients and their relatives after the study’s objectives were thoroughly explained. The two groups were matched in terms of age, gender and education level.

The inclusion criteria for patients were as follows: (a) aged between 18 and 65 years; (b) right-handed; (c) a diagnosis of schizophrenia for at least 1 year, and with no comorbid psychiatric diagnoses, including substance or alcohol use disorder; and (d) clinically stable for the past three months (i.e. no changes in symptoms severity requiring interventions such as medication adjustments or hospital admission).

The inclusion criteria for healthy controls were as follows: (a) aged between 18 and 65 years, (b) right-handed and (c) no history of past psychiatric disorder and psychotropic medication usage. Healthy controls were recruited from the local community through advertisements. All controls were screened using the Structured Clinical Interview for DSM-5, Clinical Version (SCID-5-CV)^16,17^, administered by trained psychiatrists to confirm the absence of any current or past psychiatric disorders.

The exclusion criteria for both patient and control groups were as follows: (a) presence of unstable chronic or systemic medical conditions, (b) history of loss of consciousness lasting longer than 3 min, (c) MRI findings of any lesions or space-occupying masses, or (d) any condition that contraindicates MRI scanning (e.g. pacemaker, prosthetics, pregnancy or claustrophobia).

The authors assert that all procedures contributing to this work comply with the ethical standards of the relevant national and institutional committees on human experimentation and with the Helsinki Declaration of 1975, as revised in 2013. All procedures involving human subjects/patients were approved by the Ethics Committee of Ege University (approval number 24-3T/89, approval date 13 March 2024). Trained psychiatrists interviewed all participants with the SCID-5-CV and confirmed the patients’ diagnoses. Then, the Positive and Negative Syndrome Scale (PANSS)^18,19^ was administered to assess the severity of the symptoms within the week of the MRI scan. Sociodemographic data were collected using unstructured interviews. Illness duration, daily antipsychotic dose (converted to chlorpromazine-equivalents^20^) and medication history were recorded.

### Defining treatment resistance

Treatment resistance was defined according to criteria proposed by the Treatment Response and Resistance in Psychosis (TRRIP) Working Group consensus.^21^ Patients were classified into three distinct subgroups: UTR patients, who had suboptimal clinical response (defined as a Clinical Global Impression–Severity (CGI-S)^22^ score ≥4 and PANSS score ≥4 on at least two positive or negative items, or ≥6 on one item) despite at least two previous antipsychotic trials (each at least 400 mg chlorpromazine-equivalent daily for ≥6 weeks) and subsequent clozapine treatment at a minimum dose of 300 mg/day for ≥6 weeks; Clozapine-responsive patients, who had previously failed two adequate non-clozapine antipsychotic trials (≥400 mg chlorpromazine-equivalent for ≥6 weeks each) but demonstrated significant clinical improvement following clozapine treatment (CGI-S score ≤3 and PANSS score ≤3 on all positive or negative items for ≥6 weeks); and FLR patients, who were successfully managed with first-line antipsychotics other than clozapine for ≥6 weeks, achieving clinical response (CGI-S score ≤3 and PANSS score ≤3 on all assessed items).

### MRI acquisition

During the same week with interviews, T1-weighted MRI data were collected with a Siemens Magnetom Verio Numaris/4 Syngo MR B17 and 3T MR scanner. The MRI protocol included axial sections with transverse T2-weighted sequences, obtained using the periodically rotated overlapping parallel lines with enhanced reconstruction (BLADE) technique (repetition time/echo time: 2320/117 ms, slice thickness: 5 mm, number of slices: 20, inter-slice gap: 2 mm, voxel size: 0.7 × 0.7 × 5 mm^3^, field of view (FOV): 220, number of excitations (NEX): 1, generalised autocalibrating partial parallel acquisition (GRAPPA) factor: 2); coronal sections with T2 fluid-attenuated inversion recovery, coronal (FLAIR COR) sequences (repetition time/echo time/inversion time: 9000/85/2500 ms, slice thickness: 4 mm, number of slices: 38, no inter-slice gap matrix: 192 × 256, voxel size: 1.1 × 0.9 × 4 mm^3^, FOV: 220, NEX: 1, GRAPPA factor: 2); and sagittal sections with T1-weighted 3D magnetisation-prepared rapid gradient echo (MP-RAGE) sequences (repetition time/echo time/inversion time: 1600/2.21/900 ms, flip angle: 9, slice thickness: 1 mm, number of slices: 160, no inter-slice gap matrix: 246 × 256, voxel size: 1 × 1 × 1 mm^3^, FOV: 256, NEX: 1, GRAPPA factor: 2).

### MRI preprocessing

Structural images (three-dimensional T1-weighted image) were processed with the CAT12.7 toolbox (Computational Anatomy Toolbox-Structural Brain Mapping Group, University of Jena, Jena, Germany; http://dbm.neuro.uni-jena.de/cat12/) by following the recommended pipeline in CAT12 Online Manual (http://www.neuro.uni-jena.de/cat12/CAT12-Manual.pdf), which included reorientation of images; segmentation into grey matter, white matter and cerebrospinal fluid; adjustment for partial volume effects and normalisation to Montreal Neurological Institute space with resampling to a resolution of 1.5 mm × 1.5 mm × 1.5 mm. Nonlinear modulation was then applied, and a 6-mm full-width at half-maximum Gaussian spatial smoothing kernel was used to improve the signal-to-noise ratio, and total intracranial volume (TIV) was estimated for subsequent analyses. A statistical quality assurance step was conducted with the ‘check homogeneity’ function within the CAT12.7 toolbox following the segmentation, yielding a final sample of 79 schizophrenia patients and 30 healthy controls. Statistical analyses were conducted on 23 regions of interest (ROIs), including subcortical grey matter and ventricular structures. These ROIs were defined based on the Neuromorphometrics atlas (http://www.neuromorphometrics.com/) for grey matter and cerebrospinal fluid segmentation, as implemented in the CAT12.7 toolbox.

### Hippocampal subfield volume estimation and extraction

We selected five bilateral hippocampal subfield regions that were of *a priori* interest.^12,23,24^ These included the hippocampus subfields labelled subiculum, cornus ammonis 1, cornus ammonis 2/3, cornus ammonis 4/dentate gyrus and stratum (stratum radiatum, stratum lacunosum, stratum moleculare). We used the segmentation tool implemented in CAT12.7, which uses the CoBra (Computational Brain Anatomy Laboratory at the Douglas Institute, Verdun, Canada,) atlas^25^ based on high-resolution (1 mm isotropic voxel size) images of hippocampus subfields (https://www.cobralab.ca/hippocampus-subfields).

### Statistical analysis

Statistical analyses were conducted with Jamovi (version 2.6 for macOS; The Jamovi Project, Sydney, Australia; https://www.jamovi.org) and R statistical software (version 4.4 for macOS; R Foundation for Statistical Computing, Vienna, Austria; https://www.r-project.org/). Statistical significance was set at two-tailed *P* < 0.05, with corrections for multiple comparisons applied as described below. Normality of continuous variables was evaluated with the Shapiro–Wilk test. Group differences in demographics and clinical variables were analysed with one-way analysis of variance. *Post hoc* analyses were conducted with Tukey’s honestly significant difference (HSD) test. Categorical variables were compared with chi-squared tests. Subcortical grey matter region volumes and hippocampal subfield volume were analysed with multivariate analysis of covariance (MANCOVA), controlling for age, gender and TIV, whereas ventricular volumes were analysed with age and gender as covariates, without TIV. To control for multiple testing across ROIs, the false discovery rate (FDR) correction using the Benjamini–Hochberg procedure was applied. This correction was applied across all 23 subcortical and ventricular regions. Furthermore, a separate FDR correction was applied to ten hippocampal subfield ROIs. Significant MANCOVA results (FDR-corrected *P* < 0.05) were followed up by univariate analyses of covariance, with *post hoc* pairwise comparisons conducted via Tukey’s HSD test, and Cohen’s *d* effect sizes were calculated to quantify the magnitude of between-group differences.

Spearman rank correlation analyses were used to assess associations between 23 subcortical regions and ten clinical/demographic variables. Further correlation analyses were conducted for each treatment response group. FDR correction was applied across the 230 tests for subcortical regions (23 ROIs × 10 variables) and 100 tests to correct multiple comparisons. Only FDR-adjusted *P*-values below 0.05 were considered statistically significant.

### Sensitivity analysis controlling for polypharmacy and antipsychotic medications effects

It was reported that prolonged exposure to dopamine-blocking agents can alter subcortical volumes.^26^ To examine potential confounding effects of medication dosage, a sensitivity MANCOVA was performed, including chlorpromazine-equivalent daily dose as a covariate in group comparisons of both subcortical volumes and hippocampal subfields. Additionally, partial correlation analyses were conducted within each patient subgroup, controlling for chlorpromazine-equivalent daily dose. To control for multiple testing, FDR correction with the Benjamini–Hochberg procedure was applied. Furthermore, to evaluate the potential confounding effects of adjunct antipsychotic medications, a sensitivity analysis was conducted, including only patients who were receiving antipsychotic monotherapy at the time of MRI scanning. Patients on a combination of antipsychotic treatment were excluded from this analysis. The TRS group (combined UTR and clozapine-responsive patients because of limited patient number with clozapine monotherapy in the UTR patient group) was compared with the FLR and healthy control groups, using the same statistical protocol described in the section ‘Statistical analysis’.

## Results

### Sociodemographic and clinical characteristics

Demographic comparisons revealed no significant differences among the four groups (UTR, clozapine-responsive, FLR, healthy controls) in terms of age, gender or education level (Table 1). Although the three groups did not differ in terms of age at onset and illness duration, UTR exhibited significantly higher antipsychotic dosages compared with the other schizophrenia groups, with the UTR group receiving the highest doses (mean dose±s.d.: 989.7±732.86). The UTR group also had significantly higher PANSS total, positive, negative and general psychopathology scores compared with both the clozapine-responsive and FLR groups, indicating greater symptom severity (Table 1).

**Table 1:** Participants’ demographics and clinical characteristics

Variables	UTR (*n* = 22), mean (s.d.) or *n*	Clozapine-responsive (*n* = 28), mean (s.d.) or *n*	FLR (*n* = 29), mean (s.d.) or *n*	Healthy controls (*n* = 30), mean (s.d.) or *n*	*F*/*χ* ^2^, *P*-value
Age, years	36.4 (9.17)	36.6 (10.71)	39.7 (9.76)	39.3 (8.96)	*F*(3,105) = 0.882, *P* = 0.453
Gender, male/female	18/4	17/11	18/11	17/13	χ^2^(3) = 3.92, *P* = 0.270
Education, years	12.5 (3.13)	11.8 (3.39)	12.8 (3.14)	12.7 (3.07)	*F*(3,105) = 1.29, *P* = 0.730
Age at onset, years	20.5 (5.76)	21.8 (4.48)	25.1 (8.75)	–	*F*(2,76) = 2.627, *P* = 0.083
Duration of Illness (years)	15.9 (7.78)	14.7 (8.98)	14.5 (10.06)	–	*F*(2,76) = 0.188, *P* = 0.826
Chlorpromazine antipsychotic dose equivalents, mg/day	989.7 (732.86)	548.7 (439.99)	582.4 (387.39)	–	*F*(2,76) = 5.263, *P* = 0.007^a^
PANSS total	71.3 (12.99)	43.9 (9.66)	46.0 (8.29)	–	*F*(2,76) = 52.440, *P* < 0.001^b^
PANSS positive	13.0 (6.30)	8.1 (1.51)	8.83 (2.22)	–	*F*(2,76) = 12.362, *P* < 0.001^c^
PANSS negative	25.1 (4.12)	11.7 (3.97)	13.6 (3.63)	–	*F*(2,76) = 82.811, *P* < 0.001^d^
PANSS general	33.0 (6.02)	23.1 (6.66)	23.9 (4.65)	–	*F*(2,76) = 19.184, *P* < 0.001^e^
Current antipsychotics used	Clozapine (*n* = 5), Clozapine + paliperidone (*n* = 5), Clozapine + risperidone (*n* = 4), Clozapine + amisulpride (*n* = 4), Clozapine + aripiprazole (*n* = 3), Clozapine + haloperidol (*n* = 1)	Clozapine (*n* = 13), Clozapine + paliperidone (*n* = 8), Clozapine + risperidone (*n* = 2), Clozapine + amisulpride (*n* = 3), Clozapine + zuclopenthixol (*n* = 2)	Aripiprazole (*n* = 3), Flupenthixol (*n* = 1), Risperidone (*n* = 5), Paliperidone (*n* = 1), Olanzapine (*n* = 4), Amisulpride (*n* = 1), Amisulpride+ olanzapine (*n* = 4), Paliperidone + olanzapine (*n* = 3), Aripiprazole + olanzapine (*n* = 2) Paliperidone + aripiprazole (*n* = 2) Risperidone + aripiprazole (*n* = 2) Risperidone + amisulpride (*n* = 1)	–	–

UTR, ultra-treatment-resistant; FLR, first-line antipsychotic responsive; PANSS, Positive and Negative Symptom Scale; HSD, honestly significant difference.

a .*Post hoc* Tukey HSD *P*-value: UTR > clozapine-responsive (*P* = 0.020), UTR > FLR (*P* = 0.011).

b .*Post hoc* Tukey HSD *P*-value: UTR > clozapine-responsive (*P* < 0.001), UTR > FLR (*P* < 0.001).

c .*Post hoc* Tukey HSD *P*-value: UTR > clozapine-responsive (*P* < 0.001), UTR > FLR (*P* < 0.001).

d .*Post hoc* Tukey HSD *P*-value: UTR > clozapine-responsive (*P* < 0.001), UTR > FLR (*P* < 0.001).

e .*Post hoc* Tukey HSD *P*-value: UTR > clozapine-responsive (*P* < 0.001), UTR > FLR (*P* < 0.001).

### Subcortical volume comparison

**Fig. 1:**
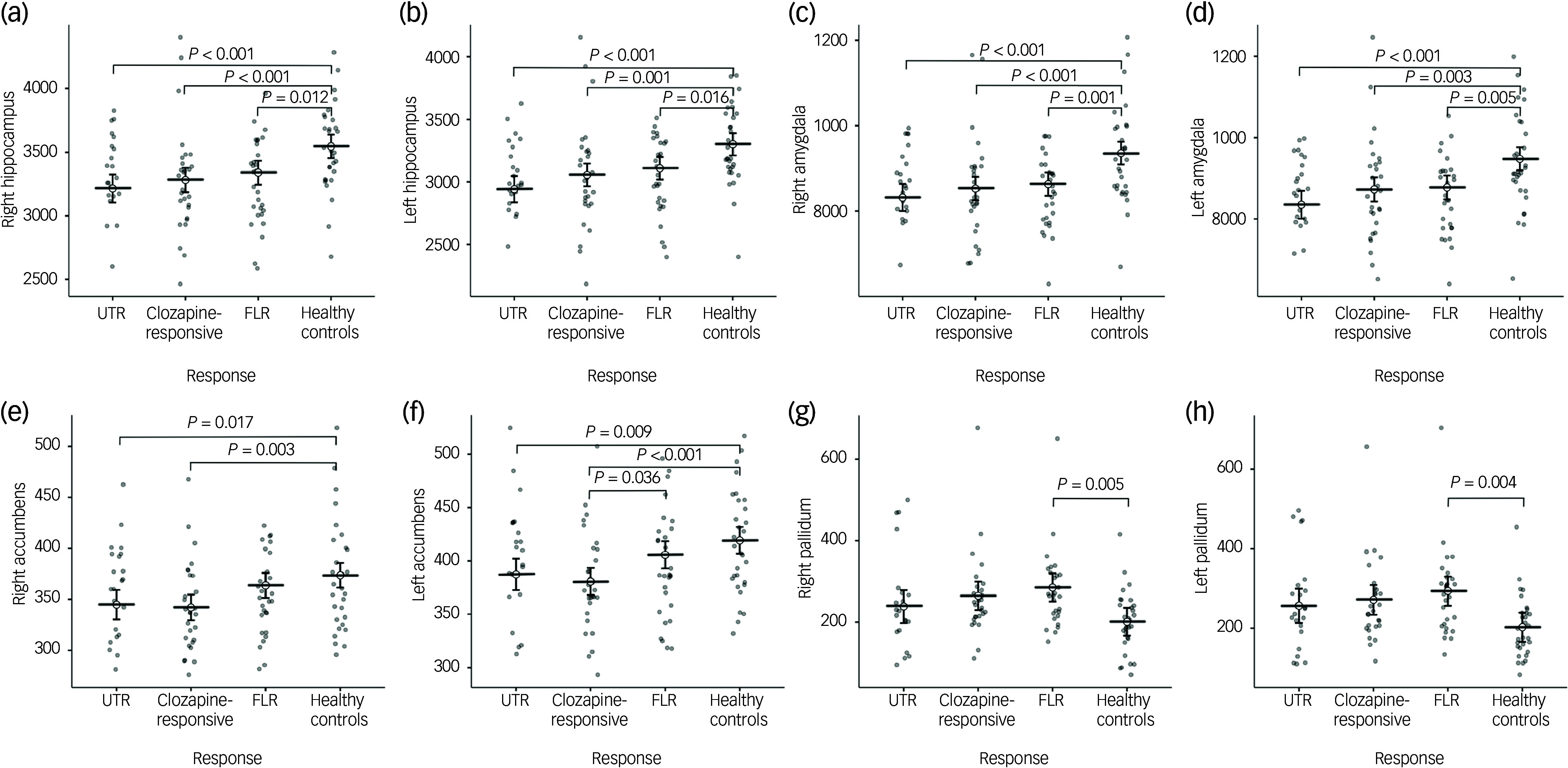
Group comparisons of mean subcortical volumes (mm³) for the right and left hippocampus (a, b), amygdala (c, d), nucleus accumbens (e, f), and pallidum (g, h) across four groups: ultra-treatment-resistant (UTR), clozapine-responsive, first-line responders (FLR) and healthy controls. Error bars represent confidence intervals. Statistical significance was assessed using post-hoc Tukey honestly significant difference tests and brackets with corresponding *P*-values indicating significant between-group differences. All volumetric values were corrected for age, gender and total intracranial volume.

**Fig. 2:**
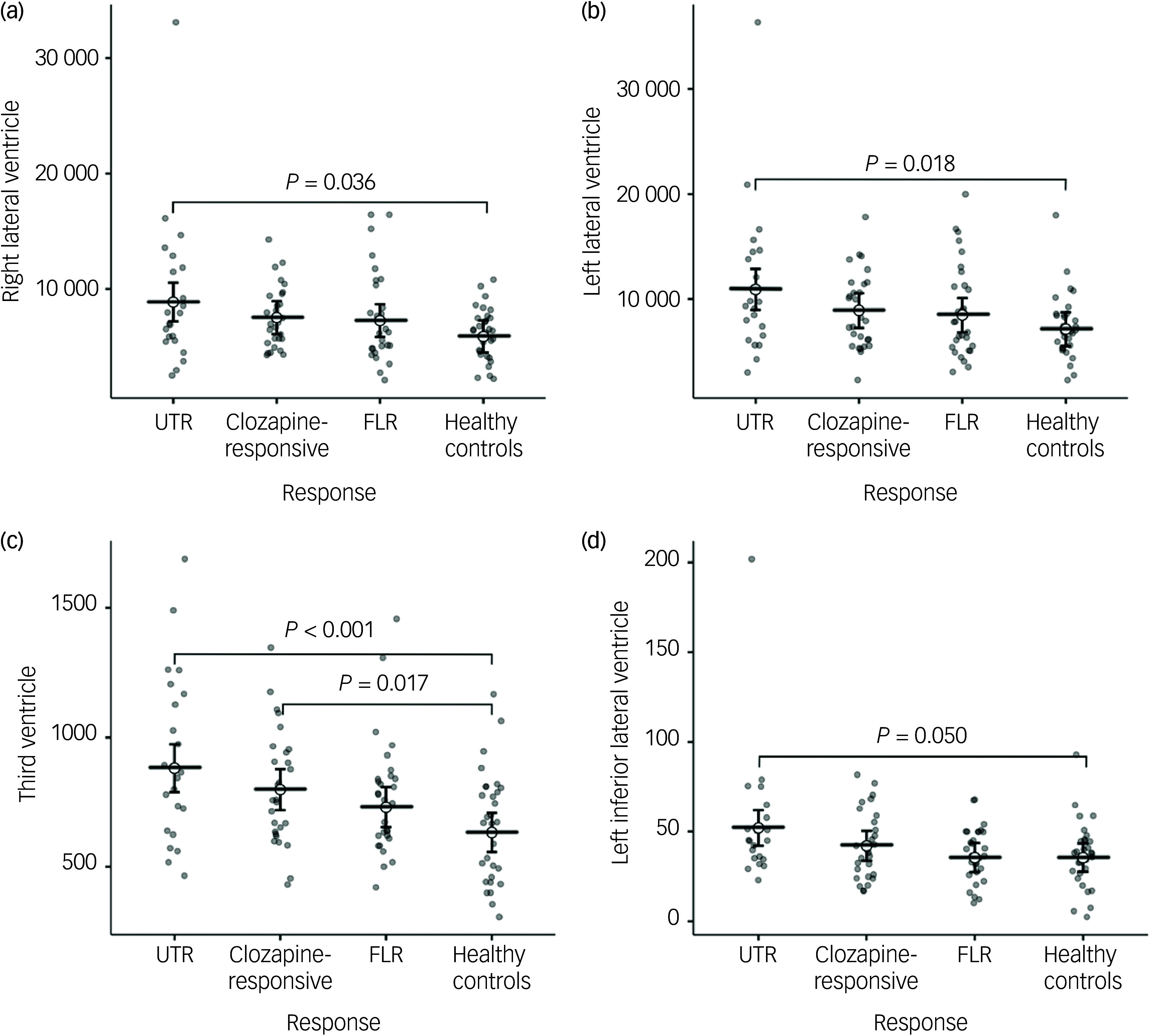
Group comparisons of mean ventricular volumes (mm³) for the right and left lateral ventricles (a, b), third ventricle (c), and left inferior lateral ventricle (d) across ultra-treatment-resistant (UTR), clozapine-responsive, first-line responders (FLR) and healthy controls. Error bars represent confidence intervals. Statistical significance was determined using post-hoc Tukey honestly significant difference tests and significant between-group differences are indicated with brackets and corresponding *P*-values. All ventricular volumes were adjusted for age and gender.

**Fig. 3:**
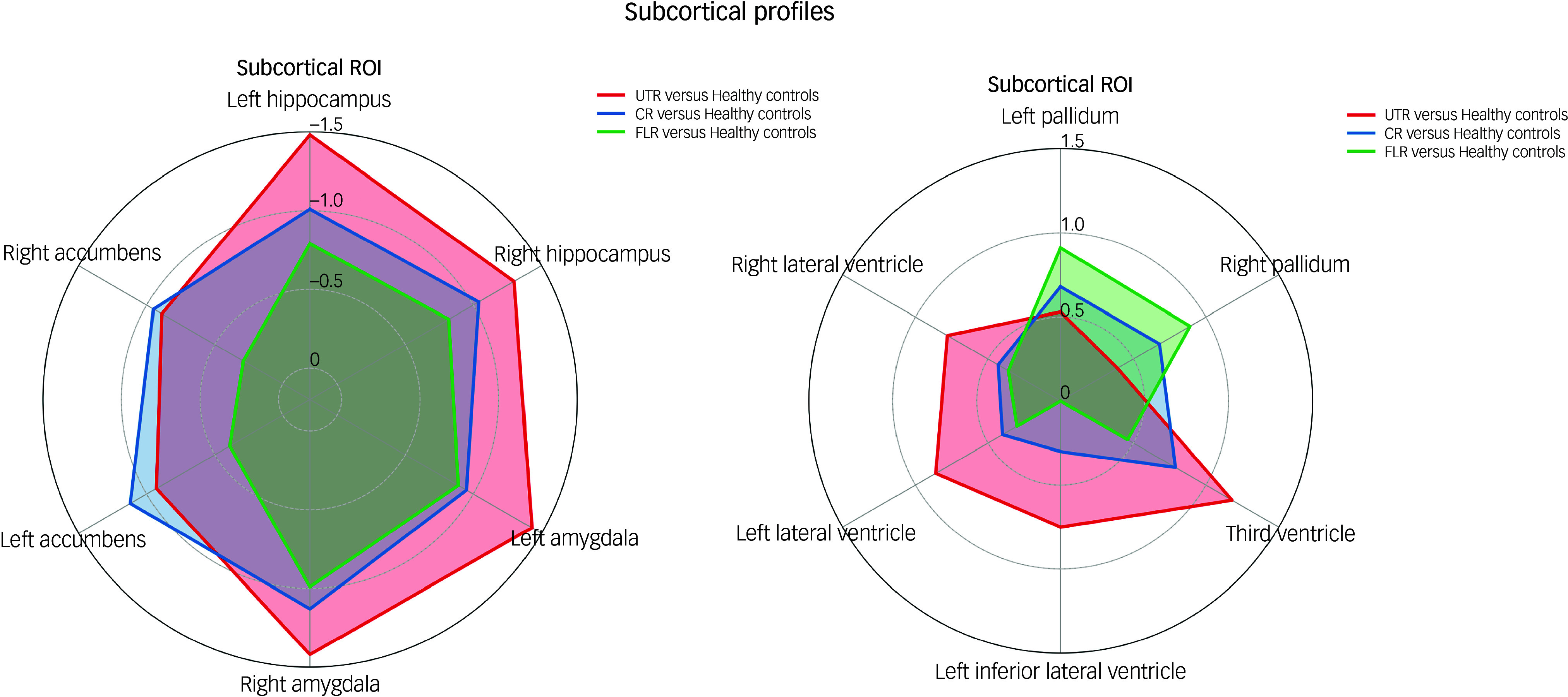
Radar plot illustrating standardised Cohen’s *d* effect sizes for each patient subgroup (ultra-treatment-resistant (UTR), clozapine-responsive and first-line responder (FLR)) relative to healthy controls across subcortical region of interest (ROI). CR, clozapine-responsive.

In the neuroanatomical comparison, a MANCOVA, controlling for age, gender and TIV, identified significant group differences in hippocampus, amygdala, accumbens and pallidal volumes (Table 2). *Post hoc* pairwise analyses indicated that treatment-resistant patients (UTR and clozapine-responsive) exhibited significantly smaller volumes in the accumbens compared with healthy controls (Fig. 1(e,f)). Furthermore, all patient groups showed smaller amygdala and hippocampal volumes relative to healthy controls (Fig. 1(a–d)). The lateral ventricular volumes and the left inferior lateral ventricle volume were significantly larger in the UTR group compared with healthy controls (Fig. 2(a,b) and (d)). Further, the third ventricle volume was larger in the treatment-resistant group (Fig. 2(c)). Notably, the pallidal volume was larger in the treatment-responsive group compared with healthy controls (Fig. 1(g,h)). There were no significant volumetric differences between the clozapine-responsive and UTR patients. Effect sizes for each patient subgroup are shown in Fig. 3.

**Table 2:** Volume differences (mm^3^) in subcortical structures between groups, controlling for age, gender and total intracranial volume

Region of interest	UTR (*n* = 22), mean (s.e.)	Clozapine-responsive (*n* = 28), mean (s.e.)	FLR (*n* = 29), mean (s.e.)	Healthy controls (*n* = 30), mean (s.e.)	*F*, *P*-value, partial *η* ^2^	FDR-corrected *P*-value
Right accumbens	344.7 (7.26)	341.9 (6.29)	363.6 (6.24)	373.6 (6.10)	*F*(3,102) = 3.861 *P* = 0.012, *η* ^2^ *P* = 0.141	*P* = 0.030^a^
Left accumbens	387.3 (7.47)	380.6 (6.47)	405.7 (6.41)	419.2 (6.27)	*F*(3,102) = 5.505, *P* = 0.002, *η* ^2^ *P* = 0.174	*P* = 0.007^b^
Right amygdala	831.5 (15.95)	852.5 (13.82)	862.9 (13.70)	935.5 (13.39)	*F*(3,102) = 8.500, *P* < 0.001, *η* ^2^ *P* = 0.232	*P* = 0.004^c^
Left amygdala	834.8 (17.27)	872.4 (14.96)	877.2 (14.83)	947.8 (14.50)	*F*(3,102) = 6.876, *P* < 0.001, *η* ^2^ *P* = 0.213	*P* = 0.004^d^
Right caudate	2270.7 (67.82)	2703.7 (58.76)	2958.7 (58.26)	2975.8 (56.94)	*F*(3,102) = 3.118, *P* = 0.029, *η* ^2^ *P* = 0.131	*P* = 0.051
Left caudate	2700.2 (65.31)	2666.1 (56.58)	2876.7 (56.10)	2914.7 (54.83)	*F*(3,102) = 2.625, *P* = 0.054, *η* ^2^ *P* = 0.119	*P* = 0.082
Right hippocampus	3213.3 (55.49)	3280.3 (48.08)	3337.2 (47.67)	3545.2 (46.59)	*F*(3,102) = 6.774, *P* < 0.001, *η* ^2^ *P* = 0.199	*P* = 0.004^e^
Left hippocampus	2941.4 (53.05)	3055.8 (45.96)	3109.0 (45.58)	3302.2 (44.54)	*F*(3,102) = 6.828, *P* < 0.001, *η* ^2^ *P* = 0.224	*P* = 0.004^f^
Right pallidum	238.1 (20.49)	264.4 (17.75)	284.9 (17.60)	200.7 (17.20)	*F*(3,102) = 3.890, *P* = 0.011, *η* ^2^ *P* = 0.113	*P* = 0.030^g^
Left pallidum	256.3 (21.80)	271.2 (18.89)	293.4 (18.73)	202.6 (18.30)	*F*(3,102) = 4.142, *P* = 0.008, *η* ^2^ *P* = 0.114	*P* = 0.026^h^
Right putamen	3240.3 (96.75)	3263.6 (83.83)	3686.5 (83.12)	3521.9 (81.24)	*F*(3,102) = 2.514, *P* = 0.063, *η* ^2^ *P* = 0.149	*P* = 0.090
Left putamen	3376.9 (104.02)	3330.27 (90.13)	3787.0 (89.36)	3587.6 (87.34)	*F*(3,102) = 2.432, *P* = 0.069, *η* ^2^ *P* = 0.131	*P* = 0.093
Right thalamus	4480.2 (120.41)	4470.1 (104.33)	4553.9 (103.45)	4826.7 (100.01)	*F*(3,102) = 2.221, *P* = 0.090, *η* ^2^ *P* = 0.070	*P* = 0.115
Left thalamus	3906.4 (116.10)	3938.4 (100.59)	4113.2 (99.74)	4361.3 (97.48)	*F*(3,102) = 2.970, *P* = 0.035, *η* ^2^ *P* = 0.108	*P* = 0.057
Right ventral diencephalon	966.6 (33.10)	1015.1 (28.68)	1069.5 (28.44)	1077.9 (27.80)	*F*(3,102) = 1.743, *P* = 0.163, *η* ^2^ *P* = 0.075	*P* = 0.197
Left ventral diencephalon	984.5 (32.95)	1049.0 (28.55)	1085.4 (28.30)	1071.7 (27.66)	*F*(3,102) = 1.002, *P* = 0.395, *η* ^2^ *P* = 0.054	*P* = 0.432
Brain stem	1372.8 (138.06)	1473.7 (119.62)	1596.5 (118.61)	1400.0 (115.92)	*F*(3,102) = 0.731, *P* = 0.535, *η* ^2^ *P* = 0.018	*P* = 0.535
Third ventricle	880.4 (46.78)	797.9 (39.86)	729.9 (39.16)	632.2 (38.25)	*F*(3,103) = 8.054, *P* < 0.001*, *η* ^2^ *P* = 0.155	*P* = 0.004^i^
Fourth ventricle	1383.0 (104.00)	1446.37 (88.61)	1323.2 (87.06)	1323.4 (85.04)	*F*(3,103) = 0.929, *P* = 0.432*, *η* ^2^ *P* = 0.012	*P* = 0.451
Right inferior lateral ventricle	94.4 (8.46)	98.5 (7.21)	79.9 (7.09)	85.2 (6.92)	*F*(3,103) = 1.271, *P* = 0.288*, *η* ^2^ *P* = 0.038	*P* = 0.331
Left inferior lateral ventricle	52.0 (4.97)	42.0 (4.17)	35.5 (4.09)	35.5 (3.99)	*F*(3,103) = 3.321, *P* = 0.023*, *η* ^2^ *P* = 0.078	*P* = 0.044^j^
Right lateral ventricle	8841.2 (849.35)	7497.7 (723.64)	7234.3 (711.42)	5862.5 (694.50)	*F*(3,103) = 3.482, *P* = 0.019*, *η* ^2^ *P* = 0.069	*P* = 0.039^k^
Left lateral ventricle	10 907.4 (987.26)	8891.5 (841.14)	8454.3 (826.48)	7111.0 (807.26)	*F*(3,103) = 3.681, *P* = 0.015*, *η* ^2^ *P* = 0.081	*P* = 0.034^l^
Total intracranial volume	1 517 263.86 (26 618.41)	1 484 330.66 (23 233.12)	1 453 790.29 (22 839.51)	1 489 735.25 (22 508.62)	*F*(3,103) = 1.255, *P* = 0.345*, *η* ^2^ *P* = 0.031	–

UTR, ultra-treatment-resistant; FLR, first-line antipsychotic responsive; FDR, false discovery rate.

a .FDR *P*-value <0.05: UTR < healthy controls (*P*_Tukey_ = 0.017, Cohen’s *d* = −0.87), clozapine-responsive< healthy controls (*P*_Tukey_ = 0.003, Cohen’s *d* = −0.95).

b .FDR *P*-value <0.05: UTR < healthy controls (*P*_Tukey_ = 0.009, Cohen’s *d* = −0.93), clozapine-responsive< healthy controls (*P*_Tukey_<0.001, Cohen’s *d* = −1.12), clozapine-responsive< FLR (*P*_Tukey_ = 0.036, Cohen’s *d* = −0.73).

c .FDR *P*-value <0.05: UTR < healthy controls (*P*_Tukey_<0.001, Cohen’s *d* = −1.42), clozapine-responsive< healthy controls (*P*_Tukey_<0.001, Cohen’s *d* = −1.13), FLR < healthy controls (*P*_Tukey_ = 0.001, Cohen’s *d* = −0.99).

d .FDR *P*-value <0.05: UTR < healthy controls (*P*_Tukey_<0.001, Cohen’s *d* = −1.43), clozapine-responsive< healthy controls (*P*_Tukey_ = 0.003, Cohen’s *d* = −0.95), FLR < healthy controls (*P*_Tukey_ = 0.005, Cohen’s *d* = −0.89).

e .FDR *P*-value <0.05: UTR < healthy controls (*P*_Tukey_<0.001, Cohen’s *d* = −1.30), clozapine-responsive< healthy controls (*P*_Tukey_<0.001, Cohen’s *d* = −1.04), FLR < healthy controls (*P*_Tukey_ = 0.012, Cohen’s *d* = −0.82).

f .FDR *P*-value <0.05: UTR < healthy controls (*P*_Tukey_<0.001, Cohen’s *d* = −1.48), clozapine-responsive< healthy controls (*P*_Tukey_ = 0.001, Cohen’s *d* = −1.01), FLR < healthy controls (*P*_Tukey_ = 0.016, Cohen’s *d* = −0.79).

g .FDR *P*-value <0.05: FLR > healthy controls (*P*_Tukey_ = 0.005, Cohen’s *d* = 0.89).

h .FDR *P*-value <0.05: FLR > healthy controls (*P*_Tukey_ = 0.004, Cohen’s *d* = 0.91).

i .FDR *P*-value <0.05: UTR > healthy controls (*P*_Tukey_<0.001, Cohen’s *d* = 1.18), clozapine-responsive> healthy controls (*P*_Tukey_ = 0.017, Cohen’s *d* = 0.79).

j .FDR *P*-value <0.05: UTR > healthy controls (*P*_Tukey_ = 0.050, Cohen’s *d* = 0.75).

k .FDR *P*-value <0.05: UTR > healthy controls (*P*_Tukey_ = 0.036, Cohen’s *d* = 0.78).

l .FDR *P*-value <0.05: UTR > healthy controls (*P*_Tukey_ = 0.018, Cohen’s *d* = 0.86).

*Controlled for age and gender.

### Hippocampal subfield volume comparison

In the neuroanatomical comparison of the hippocampal subfields, a MANCOVA, controlling for age, gender and TIV, identified significant group differences in cornus ammonis 1, subiculum, cornus ammonis 4/dentate gyrus, cornus ammonis 2/3 and stratum (Table 3). Our findings indicate that patients with TRS exhibited significantly smaller volume in the bilateral cornus ammonis 4/dentate gyrus, subiculum and stratum subfields compared with healthy controls. Additionally, UTR patients exhibited further smaller volume in the left cornus ammonis 1 and bilateral cornus ammonis 2/3 regions. Besides, FLR patients demonstrated a smaller volume limited to the bilateral cornus ammonis 4/dentate gyrus and left subiculum subfields. There were no significant volumetric differences between the clozapine-responsive and UTR patients. A detailed figure comparing hippocampal subfields and effect sizes is shown in Supplementary Figs 1 and 2 available at https://doi.org/10.1192/bjo.2025.10939.

**Table 3:** Volume differences (mm^3^) in hippocampal subfields between patient groups, controlling for age, gender and total intracranial volume

Hippocampal subfield	UTR (*n* = 22), mean (s.e.)	Clozapine-responsive (*n* = 28), mean (s.e.)	FLR (*n* = 29), mean (s.e.)	Healthy controls (*n* = 30), mean (s.e.)	*F*, *P*-value, partial *η* ^2^	FDR-corrected *P*-value
Right cornus ammonis 1	822.0 (17.64)	841.9 (16.20)	844.1 (15.64)	884.9 (16.19)	*F*(3,102) = 2.560, *P* = 0.059, *η* ^2^ *P* = 0.083	*P* = 0.059
Left cornus ammonis 1	737.9 (14.81)	771.1 (13.60)	775.3 (13.13)	815.3 (13.60)	*F*(3,102) = 4.107, *P* = 0.010, *η* ^2^ *P* = 0.151	*P* = 0.016^a^
Right subiculum	266.2 (5.51)	270.9 (5.06)	278.1 (4.88)	290.1 (5.06)	*F*(3,102) = 3.681, *P* = 0.015, *η* ^2^ *P* = 0.126	*P* = 0.021^b^
Left subiculum	272.0 (5.87)	276.5 (5.39)	279.6 (5.20)	297.6 (5.39)	*F*(3,102) = 4.617, *P* = 0.005, *η* ^2^ *P* = 0.133	*P* = 0.012^c^
Right cornus ammonis 4/dentate gyrus	647.5 (13.37)	664.9 (12.28)	669.3 (11.85)	715.2 (12.27)	*F*(3,102) = 5.603, *P* = 0.001, *η* ^2^ *P* = 0.162	*P* = 0.005^d^
Left cornus ammonis 4/dentate gyrus	637.3 (12.70)	665.7 (11.66)	671.6 (11.26)	712.8 (11.66)	*F*(3,102) = 5.572, *P* = 0.001, *η* ^2^ *P* = 0.192	*P* = 0.005^e^
Right cornus ammonis 2/3	150.9 (4.02)	153.9 (3.69)	153.7 (3.56)	165.4 (3.69)	*F*(3,102) = 3.533, *P* = 0.017, *η* ^2^ *P* = 0.098	*P* = 0.021^f^
Left cornus ammonis 2/3	111.3 (3.28)	116.3 (3.01)	117.6 (2.91)	125.9 (3.01)	*F*(3,102) = 2.987, *P* = 0.035, *η* ^2^ *P* = 0.119	*P* = 0.038^g^
Right stratum	485.8 (9.12)	491.6 (8.37)	501.9 (8.08)	529.0 (8.37)	*F*(3,102) = 5.437, *P* = 0.002, *η* ^2^ *P* = 0.156	*P* = 0.006^h^
Left stratum	501.9 (9.08)	518.4 (8.33)	526.5 (8.04)	550.8 (8.33)	*F*(3,102) = 4.388, *P* = 0.006, *η* ^2^ *P* = 0.165	*P* = 0.012^i^

UTR, ultra-treatment-resistant; FLR, first-line antipsychotic responsive; FDR, false discovery rate.

a .FDR *P*-value <0.05: UTR < healthy controls (*P*_Tukey_ < 0.001, Cohen’s *d* = −1.29).

b .FDR *P*-value <0.05: UTR < healthy controls (*P*_Tukey_ = 0.004, Cohen’s *d* = −0.99), clozapine-responsive < healthy controls (*P*_Tukey_ = 0.016, Cohen’s *d* = −0.80).

c .FDR *P*-value <0.05: UTR < healthy controls (*P*_Tukey_ = 0.004, Cohen’s *d* = −1.00), clozapine-responsive < healthy controls (*P*_Tukey_ = 0.012, Cohen’s *d* = −0.82), FLR < healthy controls (*P*_Tukey_ = 0.039, Cohen’s *d* = −0.70).

d .FDR *P*-value <0.05: UTR < healthy controls (*P*_Tukey_<0.001, Cohen’s *d* = −1.16), clozapine-responsive < healthy controls (*P*_Tukey_ = 0.008, Cohen’s *d* = −0.86), FLR < healthy controls (*P*_Tukey_ = 0.017, Cohen’s *d* = −0.79).

e .FDR *P*-value <0.05: UTR < healthy controls (*P*_Tukey_<0.001, Cohen’s *d* = −1.37), clozapine-responsive < healthy controls (*P*_Tukey_ = 0.009, Cohen’s *d* = −0.85), FLR < healthy controls (*P*_Tukey_ = 0.026, Cohen’s *d* = −0.74).

f .FDR *P*-value <0.05: UTR < healthy controls (*P*_Tukey_ = 0.024, Cohen’s *d* = −0.83).

g .FDR *P*-value <0.05: UTR < healthy controls (*P*_Tukey_ = 0.003, Cohen’s *d* = −1.02).

h .FDR *P*-value <0.05: UTR < healthy controls (*P*_Tukey_ = 0.001, Cohen’s *d* = −1.09), clozapine-responsive < healthy controls (*P*_Tukey_ = 0.003, Cohen’s *d* = −0.94).

i .FDR *P*-value <0.05: UTR < healthy controls (*P*_Tukey_<0.001, Cohen’s *d* = −1.24), clozapine-responsive < healthy controls (*P*_Tukey_ = 0.013, Cohen’s *d* = −0.82).

### Correlation analyses

After FDR correction, significant negative correlations were observed between female gender and the volumes of the accumbens, amygdala, hippocampus and third ventricle in the combined patient sample. No other significant associations were found with clinical variables, including total and subscale PANSS scores, chlorpromazine-equivalent antipsychotic dose and age at onset. Within-group analyses (FLR, clozapine-responsive and UTR) revealed no significant correlations between clinical variables and subcortical volumes. Additionally, partial correlation analyses were conducted to control for daily chlorpromazine-equivalent dosage. In these analyses, negative correlations between female gender and amygdala and hippocampal volumes persisted in the overall patient group, whereas no other significant associations were identified. Complete correlation results for each treatment-response subgroup are presented in Supplementary Tables 1–8.

### Sensitivity analysis

In the sensitivity analysis, controlling for chlorpromazine-equivalent daily dose indicates that medication dosage did not substantially influence subcortical and hippocampal subfield volumetric differences among treatment-response subgroups. Furthermore, in the second analysis, limited to patients receiving antipsychotic monotherapy, no significant differences were observed in demographic characteristics between the TRS, FLR and healthy control groups. Additionally, the patient subgroups did not differ significantly across any clinical variables. Regarding subcortical volume comparisons, both patient groups exhibited significantly smaller bilateral amygdala and right hippocampal volumes compared with healthy controls. Moreover, the TRS group demonstrated significantly lower volumes in the left hippocampus, bilateral nucleus accumbens and bilateral thalamus relative to controls. These differences remained statistically significant after FDR correction. Comprehensive clinical and neuroanatomical results from the monotherapy sensitivity analysis are presented in Supplementary Tables 9 and 10.

## Discussion

In this study, we investigated the subcortical volumes associated with different levels of treatment resistance in schizophrenia. Our findings demonstrate distinct subcortical brain structure abnormalities across treatment-resistant subgroups (UTR and clozapine responsive), FLR and healthy controls. The patient group exhibited significantly smaller subcortical volumes, particularly in the hippocampus and amygdala (Fig. 1(a–d)). Moreover, only the treatment-resistant group (UTR and clozapine-responsive) showed smaller nucleus accumbens volumes (Fig. 1(e,f)), alongside larger third and lateral ventricular volumes relative to healthy individuals (Fig. 2(a–c)). The treatment-responsive group showed larger pallidal volumes compared with healthy controls (Fig. 1(g,h)). Furthermore, hippocampal subfield analysis revealed that patients with TRS exhibited widespread smaller subfield volumes, particularly in the bilateral cornus ammonis 4/dentate gyrus, subiculum and stratum. The UTR group additionally showed smaller volumes in the left cornus ammonis 1 and bilateral cornus ammonis 2/3, whereas FLR patients showed more limited, smaller volumes.

Our observation of smaller hippocampal and amygdala volumes in patients aligns with previous neuroimaging studies that have consistently implicated limbic system abnormalities in schizophrenia.^6,9^ These limbic regions play a critical role in cognitive and emotional processes, such as memory formation, emotional regulation and stress resilience domains frequently impaired in patients with schizophrenia.^27^ Although *post hoc* comparison did not reveal significant differences between the resistant and responsive groups, treatment-resistant groups showed less mean hippocampal and amygdala volumes compared with healthy controls, which might reflect more distinct neurodegenerative processes or greater vulnerability to the chronic effects of psychosis in treatment-resistant populations.^13,28^ Our findings are consistent with those reported by Liu et al, who observed similar, smaller volumes in the hippocampus, nucleus accumbens and putamen, as well as increased ventricular regions, among patients with TRS compared with those in early-stage schizophrenia, further supporting the hypothesis of distinct subcortical structural pathology in treatment resistance.^13^ These findings align with our results, which similarly demonstrate structural abnormalities in subcortical regions among patients with varying treatment-response profiles.

Furthermore, the significantly smaller nucleus accumbens volumes observed in UTR and clozapine-responsive groups, but not in the FLR group, compared with healthy controls, suggest abnormalities within dopaminergic reward circuits, which may contribute to diminished antipsychotic responsiveness. Previous studies highlight the accumbens/ventral striatum as central in reward processing, motivation and response to antipsychotic medication.^29,30^ Dysfunction within this circuitry may impede treatment efficacy, reflecting impaired dopaminergic signalling pathways specifically implicated in the pathophysiology of treatment resistance.

Another key distinction between the treatment-responsive and resistant groups was pallidal volumes. The FLR group, not the treatment-resistant group, showed larger pallidal volumes compared with controls. This larger volume has been constantly shown in prior literature.^8,9^ Similarly, Kim et al demonstrated larger pallidal and striatal volumes; they interpreted this finding as presynaptic dopamine synthesis and release capacity in these regions associated with the first-line treatment response, which is not observed in treatment-resistant patients.^31^ Furthermore, pallidal and striatal regions are known to exhibit volume increases in response to antipsychotic exposure, especially with first- and second-generation agents.^32,33^ Conversely, clozapine has been associated with smaller subcortical volumes relative to other antipsychotics. Jørgensen et al reported reduced pallidal and striatal volumes in clozapine-treated patients compared with those on non-clozapine medications.^34^ Similarly, a recent study by Chen et al demonstrated a positive correlation between treatment improvement and pallidal volume, alongside a negative correlation with amygdala volume.^35^ These findings suggest that pallidal volume may serve as a potential biomarker of antipsychotic response, whereas clozapine’s comparatively attenuated impact on subcortical structures may explain the lack of such enlargement in patients with TRS.

Furthermore, hippocampal subfield volumes differed across schizophrenia treatment-response profiles, with the most extensive smaller volumes observed in clozapine-resistant (UTR) patients. Patients with TRS (UTR and clozapine-responsive) showed smaller bilateral cornus ammonis 4/dentate gyrus, subiculum and stratum volumes, whereas UTR patients exhibited additional smaller left cornus ammonis 1 and bilateral cornus ammonis 2/3 volumes. FLR patients demonstrated a more restricted pattern, limited to the bilateral cornus ammonis 4/dentate gyrus and left subiculum. Our findings align with the emerging evidence that structural alterations are not uniformly distributed across the hippocampus, but are concentrated in specific subfields.^10,12^ Reductions in cornus ammonis 4/dentate gyrus were observed even in the early stage of the illness,^14,36–38^ which is exhibited across the patient groups in our sample. Furthermore, the more widespread hippocampal subfield alterations in UTR compared with clozapine-responsive patients suggest that the degree and distribution of hippocampal pathology may relate to the severity and refractoriness of the illness. In line with these findings, Fortier et al reported widespread reductions in hippocampal subfield volumes, specifically in the left subiculum, left cornus ammonis 4/dentate gyrus, and bilateral cornus ammonis 1, in patients with TRS compared with those who were treatment-responsive.^10^ Furthermore, Briend et al found that smaller cornus ammonis 1 and subiculum volumes were associated with a longer duration of untreated illness.^14^ Moreover, Qi et al demonstrated that reduced volumes in the dentate gyrus, cornus ammonis 1 and cornus ammonis 2/3 predicted poorer treatment adherence.^39^ Such a pattern may reflect glutamatergic dysfunction and excitotoxicity,^12,23,36,40^ which have been proposed as alternative or complementary mechanisms to dopaminergic dysregulation in TRS pathophysiology.^41^ Our findings also underscore the potential value of hippocampal subfield volumetry as a candidate marker for patient stratification. However, caution is warranted in interpreting these results. The cross-sectional design precludes causal inferences about whether subfield changes are antecedents or consequences of treatment resistance.

Lateral and third ventricular volumes were significantly enlarged in the treatment-resistant groups. Ventricular enlargement is a well-established neuroanatomical hallmark of schizophrenia, and is commonly interpreted as a marker of global brain atrophy, neurodevelopmental disruption or disease progression.^42^ Our findings suggest that this enlargement may be more pronounced among treatment-resistant individuals, in line with previous reports.^13,28^ This pattern may reflect more severe or accelerated neuroprogressive processes in this subgroup, potentially associated with poorer functional outcomes and diminished treatment responsiveness.

Correlation analyses revealed associations between subcortical volumes and gender, but only in the combined patient sample. Female gender showed negative correlations with the volumes of the hippocampus, amygdala, accumbens and third ventricle, suggesting possible gender-related structural differences within limbic circuits. However, these associations were not replicated within treatment-response subgroups (FLR, clozapine-responsive or UTR) and did not remain significant for other clinical variables, including age, illness duration, chlorpromazine-equivalent dose and PANSS scores. Given the cross-sectional nature of the study and the absence of consistent findings across analyses, these correlations should be interpreted with caution, and considered exploratory rather than conclusive.

### Strengths and limitations

The main strength of this study lies in its well-defined clinical sample consisting solely of patients with schizophrenia. Another strength is the use of detailed volumetric analyses covering both subcortical regions and hippocampal subfields. Finally, the inclusion of clinically distinct treatment-response subgroups enables a more precise examination of the neuroanatomical patterns associated with treatment resistance.

Despite these strengths, certain limitations should be acknowledged. First, its cross-sectional nature limits our ability to determine causal relationships or longitudinal progression of neuroanatomical changes, particularly for clozapine, after the initiation of the clozapine progressive subcortical volume reduction reported.^43^ Differences in the type and duration of prior antipsychotic treatment may contribute to heterogeneity across patients. The effects of such exposure on brain volumes remain unclear and may vary between individuals. Furthermore, symptom severity was assessed only at the time of imaging, and prospective longitudinal assessments were not conducted. This cross-sectional design also limits the ability to infer causal relationships between treatment type and structural brain measures. Future longitudinal studies could better elucidate temporal trajectories of brain volume alterations relative to treatment response. Second, because of the unavailability of clozapine blood level measurements in our clinic, the classification of UTR and clozapine-responsive groups was based solely on reported daily dosages. As a result, we may have missed cases with subtherapeutic clozapine levels despite adequate dosing. However, all patients included in the UTR group received clozapine at daily doses equal to or exceeding 300 mg/day for at least 6 weeks, consistent with the TRRIP Working Group criteria for adequate clozapine trials. Third, patients with schizophrenia, particularly TRS, often require adjunctive medications such as other antipsychotics, antidepressants, mood stabilisers and benzodiazepines.^44^ Although these medications may influence brain volume,^45,46^ their effects were not systematically controlled in this study. To assess the potential confounding effects of adjunctive antipsychotics, we conducted a sensitivity analysis including only patients on antipsychotic monotherapy. In this analysis, the TRS group was compared with FLR and healthy controls. These results indicate that the structural differences observed in the main analysis persist even after excluding patients receiving adjunctive antipsychotic medications, suggesting that these volumetric reductions represent core neurobiological alterations associated with treatment resistance.

### Implications

In conclusion, our findings indicate that distinct subcortical and hippocampal subfield volumetric profiles distinguish schizophrenia subgroups defined by treatment responsiveness. Although smaller hippocampal and amygdala volumes seem to represent shared neuroanatomical features, reduced accumbens and enlarged pallidal volumes differentiate treatment-resistant and treatment-responsive patterns, respectively. These dissociable structural signatures underscore the heterogeneity of schizophrenia and point toward limbic–striatal circuit dysfunction as a potential substrate of poor antipsychotic response. Future integration of such imaging-derived biomarkers with clinical and molecular measures could facilitate individualised treatment strategies and support early identification of patients at risk for treatment resistance.

## Supporting information

Sungur et al. supplementary material Sungur et al. supplementary material

## Data Availability

The data-sets used and/or analysed during the current study are available from the corresponding author, A.S.G., on reasonable request.

## Supplementary material

The supplementary material is available online at https://doi.org/10.1192/bjo.2025.10939
