# Supplementary material for: Distinct subcortical neuroanatomic profiles of treatment-resistant schizophrenia: structural magnetic resonance imaging study
Source: BJPsych Open. 2026 Jan 12;12(1):e36. doi: 10.1192/bjo.2025.10939 (PMC12835719; doi:10.1192/bjo.2025.10939)
Supplement: Sungur et al. supplementary material [file S2056472425109393sup001.docx]

Supplementary Table 1. Correlation between demographic and clinical characteristics and mean subcortical volumes in all participants

| Region of Interest | Sex | Age | Education | Age of Onset | Duration of Illness | CPZ Daily Dose | T-PANSS | P-PANSS | N-PANSS | G-PANSS |
| --- | --- | --- | --- | --- | --- | --- | --- | --- | --- | --- |
| Third Ventricle | ρ=-0.390*, q=0.018 | ρ=0.337, q=0.068 | ρ=0.076, q=0.719 | ρ=0.096, q=0.659 | ρ=0.306, q=0.130 | ρ=0.252, q=0.182 | ρ=0.088, q=0.676 | ρ=0.071, q=0.730 | ρ=0.012, q=0.977 | ρ=0.089, q=0.676 |
| Fourth Ventricle | ρ=-0.108, q=0.652 | ρ=-0.296, q=0.133 | ρ=0.112, q=0.652 | ρ=-0.199, q=0.369 | ρ=-0.181, q=0.425 | ρ=0.257, q=0.172 | ρ=0.128, q=0.602 | ρ=0.092, q=0.674 | ρ=0.089, q=0.676 | ρ=0.177, q=0.430 |
| Right Accumbens | ρ=-0.359*, q=0.038 | ρ=-0.174, q=0.431 | ρ=0.214, q=0.332 | ρ=-0.213, q=0.332 | ρ=-0.041, q=0.848 | ρ=0.104, q=0.652 | ρ=0.075, q=0.719 | ρ=0.041, q=0.847 | ρ=0.025, q=0.929 | ρ=0.049, q=0.822 |
| Left Accumbens | ρ=-0.369*, q=0.032 | ρ=-0.146, q=0.546 | ρ=0.186, q=0.413 | ρ=-0.204, q=0.353 | ρ=-0.002, q=0.988 | ρ=0.206, q=0.347 | ρ=0.084, q=0.676 | ρ=0.066, q=0.744 | ρ=0.067, q=0.744 | ρ=0.019, q=0.958 |
| Right Amygdala | ρ=-0.447*, q=0.003 | ρ=-0.199, q=0.369 | ρ=0.103, q=0.652 | ρ=-0.177, q=0.430 | ρ=-0.003, q=0.982 | ρ=0.173, q=0.431 | ρ=0.192, q=0.392 | ρ=0.136, q=0.573 | ρ=0.121, q=0.615 | ρ=0.176, q=0.430 |
| Left Amygdala | ρ=-0.441*, q=0.003 | ρ=-0.156, q=0.506 | ρ=0.154, q=0.514 | ρ=-0.193, q=0.389 | ρ=0.037, q=0.865 | ρ=0.149, q=0.540 | ρ=0.113, q=0.652 | ρ=0.061, q=0.761 | ρ=0.049, q=0.822 | ρ=0.094, q=0.665 |
| Brain Stem | ρ=0.005, q=0.977 | ρ=0.187, q=0.413 | ρ=0.117, q=0.633 | ρ=-0.005, q=0.977 | ρ=0.143, q=0.562 | ρ=0.046, q=0.838 | ρ=-0.292, q=0.139 | ρ=-0.038, q=0.864 | ρ=-0.257, q=0.172 | ρ=-0.303, q=0.130 |
| Right Caudate | ρ=-0.275, q=0.172 | ρ=-0.241, q=0.217 | ρ=0.149, q=0.540 | ρ=-0.239, q=0.221 | ρ=-0.055, q=0.794 | ρ=0.148, q=0.540 | ρ=0.110, q=0.652 | ρ=0.110, q=0.652 | ρ=0.017, q=0.958 | ρ=0.099, q=0.658 |
| Left Caudate | ρ=-0.267, q=0.172 | ρ=-0.268, q=0.172 | ρ=0.181, q=0.425 | ρ=-0.264, q=0.172 | ρ=-0.074, q=0.720 | ρ=0.170, q=0.448 | ρ=0.089, q=0.676 | ρ=0.086, q=0.676 | ρ=0.006, q=0.977 | ρ=0.066, q=0.744 |
| Right Hippocampus | ρ=-0.437*, q=0.003 | ρ=-0.123, q=0.609 | ρ=0.115, q=0.639 | ρ=-0.167, q=0.449 | ρ=0.009, q=0.977 | ρ=0.136, q=0.573 | ρ=0.167, q=0.449 | ρ=0.105, q=0.652 | ρ=0.098, q=0.658 | ρ=0.163, q=0.461 |
| Left Hippocampus | ρ=-0.468*, q=0.003 | ρ=-0.146, q=0.546 | ρ=0.137, q=0.573 | ρ=-0.165, q=0.453 | ρ=-0.030, q=0.900 | ρ=0.072, q=0.730 | ρ=0.120, q=0.615 | ρ=0.123, q=0.609 | ρ=0.018, q=0.958 | ρ=0.134, q=0.573 |
| Right Inferior Lateral Ventricle | ρ=-0.168, q=0.449 | ρ=0.134, q=0.573 | ρ=0.050, q=0.819 | ρ=0.126, q=0.606 | ρ=0.165, q=0.453 | ρ=0.083, q=0.679 | ρ=-0.102, q=0.653 | ρ=-0.134, q=0.573 | ρ=-0.084, q=0.676 | ρ=-0.087, q=0.676 |
| Left Inferior Lateral Ventricle | ρ=-0.301, q=0.130 | ρ=0.112, q=0.652 | ρ=0.009, q=0.977 | ρ=0.151, q=0.540 | ρ=0.042, q=0.847 | ρ=0.159, q=0.496 | ρ=0.104, q=0.652 | ρ=0.046, q=0.838 | ρ=0.030, q=0.900 | ρ=0.107, q=0.652 |
| Right Lateral Ventricle | ρ=-0.265, q=0.172 | ρ=0.089, q=0.676 | ρ=0.142, q=0.563 | ρ=0.008, q=0.977 | ρ=0.086, q=0.676 | ρ=0.184, q=0.424 | ρ=0.009, q=0.977 | ρ=0.006, q=0.977 | ρ=-0.068, q=0.740 | ρ=0.014, q=0.977 |
| Left Lateral Ventricle | ρ=-0.226, q=0.276 | ρ=0.088, q=0.676 | ρ=0.110, q=0.652 | ρ=0.017, q=0.958 | ρ=0.069, q=0.740 | ρ=0.193, q=0.389 | ρ=0.057, q=0.789 | ρ=-0.012, q=0.977 | ρ=-0.011, q=0.977 | ρ=0.044, q=0.840 |
| Right Pallidum | ρ=-0.125, q=0.606 | ρ=-0.075, q=0.719 | ρ=0.018, q=0.958 | ρ=0.012, q=0.977 | ρ=-0.097, q=0.658 | ρ=0.275, q=0.172 | ρ=-0.097, q=0.658 | ρ=0.096, q=0.660 | ρ=-0.133, q=0.578 | ρ=-0.095, q=0.663 |
| Left Pallidum | ρ=-0.086, q=0.676 | ρ=0.057, q=0.789 | ρ=0.030, q=0.900 | ρ=0.122, q=0.609 | ρ=-0.069, q=0.740 | ρ=0.207, q=0.347 | ρ=-0.075, q=0.719 | ρ=0.129, q=0.601 | ρ=-0.085, q=0.676 | ρ=-0.106, q=0.652 |
| Right Putamen | ρ=-0.255, q=0.172 | ρ=-0.236, q=0.225 | ρ=0.106, q=0.652 | ρ=-0.197, q=0.373 | ρ=-0.104, q=0.652 | ρ=0.175, q=0.431 | ρ=0.080, q=0.702 | ρ=0.100, q=0.658 | ρ=0.005, q=0.977 | ρ=0.089, q=0.676 |
| Left Putamen | ρ=-0.268, q=0.172 | ρ=-0.207, q=0.347 | ρ=0.117, q=0.633 | ρ=-0.177, q=0.430 | ρ=-0.067, q=0.744 | ρ=0.237, q=0.225 | ρ=0.135, q=0.573 | ρ=0.125, q=0.606 | ρ=0.074, q=0.720 | ρ=0.102, q=0.653 |
| Right Thalamus | ρ=-0.181, q=0.425 | ρ=-0.207, q=0.347 | ρ=-0.078, q=0.707 | ρ=-0.260, q=0.172 | ρ=-0.007, q=0.977 | ρ=0.135, q=0.573 | ρ=0.035, q=0.878 | ρ=0.028, q=0.909 | ρ=0.064, q=0.744 | ρ=-0.055, q=0.794 |
| Left Thalamus | ρ=-0.106, q=0.652 | ρ=-0.326, q=0.086 | ρ=-0.138, q=0.573 | ρ=-0.259, q=0.172 | ρ=-0.144, q=0.556 | ρ=0.128, q=0.602 | ρ=0.007, q=0.977 | ρ=-0.022, q=0.946 | ρ=0.062, q=0.758 | ρ=-0.054, q=0.796 |
| Right Ventral Diencephalon | ρ=-0.191, q=0.392 | ρ=0.099, q=0.658 | ρ=0.223, q=0.284 | ρ=-0.046, q=0.838 | ρ=0.099, q=0.658 | ρ=0.042, q=0.847 | ρ=-0.289, q=0.140 | ρ=-0.065, q=0.744 | ρ=-0.301, q=0.130 | ρ=-0.274, q=0.172 |
| Left Ventral Diencephalon | ρ=-0.203, q=0.355 | ρ=0.123, q=0.609 | ρ=0.173, q=0.431 | ρ=-0.031, q=0.900 | ρ=0.104, q=0.652 | ρ=0.018, q=0.958 | ρ=-0.255, q=0.172 | ρ=-0.041, q=0.847 | ρ=-0.267, q=0.172 | ρ=-0.243, q=0.213 |

*FDR corrected p-values <0.05. CPZ: Chlorpromazine equivalent, T-PANSS: Positive and Negative Symptom Scale total score, P-PANSS: Positive and Negative Symptom Scale positive subscale score, N-PANSS: Positive and Negative Symptom Scale negative subscale score, G-PANSS: Positive and Negative Symptom Scale general subscale score.

Supplementary Table 2. Correlation between demographic and clinical characteristics and mean subcortical volumes in first-line responders

| Region of Interest | Sex | Age | Education | Age of Onset | Duration of Illness | CPZ Daily Dose | T-PANSS | P-PANSS | N-PANSS | G-PANSS |
| --- | --- | --- | --- | --- | --- | --- | --- | --- | --- | --- |
| Third Ventricle | ρ=-0.170, q=0.910 | ρ=0.541, q=0.188 | ρ=-0.129, q=0.969 | ρ=0.138, q=0.969 | ρ=0.278, q=0.724 | ρ=0.545, q=0.188 | ρ=0.051, q=0.993 | ρ=-0.073, q=0.993 | ρ=0.015, q=0.993 | ρ=-0.040, q=0.993 |
| Fourth Ventricle | ρ=0.076, q=0.993 | ρ=-0.243, q=0.798 | ρ=0.107, q=0.993 | ρ=-0.147, q=0.937 | ρ=-0.264, q=0.735 | ρ=0.170, q=0.910 | ρ=0.045, q=0.993 | ρ=-0.209, q=0.853 | ρ=-0.047, q=0.993 | ρ=0.166, q=0.912 |
| Right Accumbens | ρ=-0.357, q=0.646 | ρ=-0.148, q=0.937 | ρ=0.077, q=0.993 | ρ=-0.393, q=0.577 | ρ=0.179, q=0.908 | ρ=0.095, q=0.993 | ρ=-0.202, q=0.853 | ρ=0.020, q=0.993 | ρ=-0.294, q=0.721 | ρ=-0.281, q=0.724 |
| Left Accumbens | ρ=-0.416, q=0.517 | ρ=-0.002, q=0.993 | ρ=0.009, q=0.993 | ρ=-0.355, q=0.646 | ρ=0.274, q=0.732 | ρ=0.233, q=0.842 | ρ=-0.183, q=0.897 | ρ=-0.074, q=0.993 | ρ=-0.186, q=0.891 | ρ=-0.341, q=0.671 |
| Right Amygdala | ρ=-0.374, q=0.646 | ρ=-0.312, q=0.721 | ρ=-0.130, q=0.969 | ρ=-0.271, q=0.735 | ρ=0.017, q=0.993 | ρ=0.200, q=0.853 | ρ=0.050, q=0.993 | ρ=0.100, q=0.993 | ρ=0.028, q=0.993 | ρ=-0.031, q=0.993 |
| Left Amygdala | ρ=-0.450, q=0.410 | ρ=-0.288, q=0.724 | ρ=-0.134, q=0.969 | ρ=-0.431, q=0.462 | ρ=0.131, q=0.969 | ρ=0.194, q=0.856 | ρ=0.046, q=0.993 | ρ=0.070, q=0.993 | ρ=0.025, q=0.993 | ρ=-0.041, q=0.993 |
| Brain Stem | ρ=-0.068, q=0.993 | ρ=0.293, q=0.721 | ρ=0.116, q=0.993 | ρ=0.050, q=0.993 | ρ=0.153, q=0.937 | ρ=0.175, q=0.910 | ρ=-0.223, q=0.844 | ρ=0.162, q=0.931 | ρ=-0.229, q=0.844 | ρ=-0.203, q=0.853 |
| Right Caudate | ρ=-0.221, q=0.844 | ρ=-0.278, q=0.724 | ρ=-0.147, q=0.937 | ρ=-0.393, q=0.577 | ρ=0.109, q=0.993 | ρ=0.160, q=0.935 | ρ=-0.136, q=0.969 | ρ=0.023, q=0.993 | ρ=-0.224, q=0.844 | ρ=-0.257, q=0.770 |
| Left Caudate | ρ=-0.238, q=0.821 | ρ=-0.313, q=0.721 | ρ=-0.105, q=0.993 | ρ=-0.461, q=0.404 | ρ=0.120, q=0.993 | ρ=0.265, q=0.735 | ρ=-0.091, q=0.993 | ρ=0.053, q=0.993 | ρ=-0.206, q=0.853 | ρ=-0.230, q=0.844 |
| Right Hippocampus | ρ=-0.467, q=0.404 | ρ=0.010, q=0.993 | ρ=-0.131, q=0.969 | ρ=-0.296, q=0.721 | ρ=0.282, q=0.724 | ρ=0.112, q=0.993 | ρ=0.015, q=0.993 | ρ=-0.006, q=0.993 | ρ=0.097, q=0.993 | ρ=-0.148, q=0.937 |
| Left Hippocampus | ρ=-0.582, q=0.188 | ρ=-0.078, q=0.993 | ρ=0.003, q=0.993 | ρ=-0.254, q=0.782 | ρ=0.113, q=0.993 | ρ=-0.075, q=0.993 | ρ=0.151, q=0.937 | ρ=0.121, q=0.993 | ρ=0.095, q=0.993 | ρ=0.002, q=0.993 |
| Right Inferior Lateral Ventricle | ρ=-0.501, q=0.323 | ρ=-0.153, q=0.937 | ρ=0.002, q=0.993 | ρ=0.002, q=0.993 | ρ=-0.063, q=0.993 | ρ=0.299, q=0.721 | ρ=-0.200, q=0.853 | ρ=-0.178, q=0.908 | ρ=-0.062, q=0.993 | ρ=-0.118, q=0.993 |
| Left Inferior Lateral Ventricle | ρ=-0.459, q=0.404 | ρ=-0.044, q=0.993 | ρ=-0.062, q=0.993 | ρ=0.120, q=0.993 | ρ=-0.218, q=0.853 | ρ=0.108, q=0.993 | ρ=-0.062, q=0.993 | ρ=-0.094, q=0.993 | ρ=-0.074, q=0.993 | ρ=-0.098, q=0.993 |
| Right Lateral Ventricle | ρ=-0.195, q=0.856 | ρ=0.084, q=0.993 | ρ=0.035, q=0.993 | ρ=0.054, q=0.993 | ρ=-0.069, q=0.993 | ρ=0.295, q=0.721 | ρ=0.101, q=0.993 | ρ=0.069, q=0.993 | ρ=-0.151, q=0.937 | ρ=0.083, q=0.993 |
| Left Lateral Ventricle | ρ=-0.076, q=0.993 | ρ=0.029, q=0.993 | ρ=-0.136, q=0.969 | ρ=0.008, q=0.993 | ρ=-0.039, q=0.993 | ρ=0.327, q=0.688 | ρ=0.084, q=0.993 | ρ=-0.009, q=0.993 | ρ=-0.130, q=0.969 | ρ=-0.002, q=0.993 |
| Right Pallidum | ρ=-0.204, q=0.853 | ρ=0.171, q=0.910 | ρ=0.222, q=0.844 | ρ=0.121, q=0.993 | ρ=0.004, q=0.993 | ρ=0.365, q=0.646 | ρ=-0.059, q=0.993 | ρ=0.246, q=0.788 | ρ=-0.332, q=0.671 | ρ=-0.043, q=0.993 |
| Left Pallidum | ρ=-0.102, q=0.993 | ρ=0.310, q=0.721 | ρ=0.049, q=0.993 | ρ=0.280, q=0.724 | ρ=-0.070, q=0.993 | ρ=0.184, q=0.896 | ρ=0.077, q=0.993 | ρ=0.332, q=0.671 | ρ=-0.089, q=0.993 | ρ=0.005, q=0.993 |
| Right Putamen | ρ=-0.246, q=0.788 | ρ=-0.227, q=0.844 | ρ=-0.018, q=0.993 | ρ=-0.269, q=0.735 | ρ=0.024, q=0.993 | ρ=0.095, q=0.993 | ρ=0.022, q=0.993 | ρ=0.023, q=0.993 | ρ=-0.087, q=0.993 | ρ=-0.080, q=0.993 |
| Left Putamen | ρ=-0.246, q=0.788 | ρ=-0.170, q=0.910 | ρ=-0.042, q=0.993 | ρ=-0.266, q=0.735 | ρ=0.079, q=0.993 | ρ=0.043, q=0.993 | ρ=0.042, q=0.993 | ρ=-0.024, q=0.993 | ρ=-0.008, q=0.993 | ρ=-0.093, q=0.993 |
| Right Thalamus | ρ=-0.340, q=0.671 | ρ=0.048, q=0.993 | ρ=-0.206, q=0.853 | ρ=-0.335, q=0.671 | ρ=0.360, q=0.646 | ρ=-0.024, q=0.993 | ρ=-0.168, q=0.910 | ρ=-0.157, q=0.937 | ρ=-0.083, q=0.993 | ρ=-0.315, q=0.721 |
| Left Thalamus | ρ=-0.246, q=0.788 | ρ=-0.039, q=0.993 | ρ=-0.117, q=0.993 | ρ=-0.429, q=0.462 | ρ=0.369, q=0.646 | ρ=-0.014, q=0.993 | ρ=-0.190, q=0.874 | ρ=-0.159, q=0.935 | ρ=-0.088, q=0.993 | ρ=-0.305, q=0.721 |
| Right Ventral Diencephalon | ρ=-0.404, q=0.574 | ρ=0.214, q=0.853 | ρ=0.350, q=0.659 | ρ=0.030, q=0.993 | ρ=0.067, q=0.993 | ρ=0.010, q=0.993 | ρ=-0.129, q=0.969 | ρ=0.197, q=0.856 | ρ=-0.305, q=0.721 | ρ=-0.034, q=0.993 |
| Left Ventral Diencephalon | ρ=-0.382, q=0.624 | ρ=0.289, q=0.724 | ρ=0.206, q=0.853 | ρ=0.020, q=0.993 | ρ=0.168, q=0.910 | ρ=0.032, q=0.993 | ρ=-0.051, q=0.993 | ρ=0.199, q=0.853 | ρ=-0.210, q=0.853 | ρ=-0.020, q=0.993 |

*FDR corrected p-values <0.05. CPZ: Chlorpromazine equivalent, T-PANSS: Positive and Negative Symptom Scale total score, P-PANSS: Positive and Negative Symptom Scale positive subscale score, N-PANSS: Positive and Negative Symptom Scale negative subscale score, G-PANSS: Positive and Negative Symptom Scale general subscale score.

Supplementary Table 3. Correlation between demographic and clinical characteristics and mean subcortical volumes in clozapine responders

| Region of Interest | Sex | Age | Education | Age of Onset | Duration of Illness | CPZ Daily Dose | T-PANSS | P-PANSS | N-PANSS | G-PANSS |
| --- | --- | --- | --- | --- | --- | --- | --- | --- | --- | --- |
| Third Ventricle | ρ=-0.521, q=0.179 | ρ=0.262, q=0.558 | ρ=0.197, q=0.646 | ρ=0.013, q=0.969 | ρ=0.378, q=0.398 | ρ=0.225, q=0.599 | ρ=-0.027, q=0.953 | ρ=0.212, q=0.609 | ρ=-0.294, q=0.513 | ρ=0.094, q=0.808 |
| Fourth Ventricle | ρ=-0.240, q=0.567 | ρ=-0.291, q=0.516 | ρ=0.050, q=0.909 | ρ=-0.385, q=0.382 | ρ=-0.011, q=0.974 | ρ=0.312, q=0.511 | ρ=0.268, q=0.557 | ρ=0.560, q=0.149 | ρ=0.173, q=0.677 | ρ=0.219, q=0.609 |
| Right Accumbens | ρ=-0.249, q=0.558 | ρ=-0.339, q=0.493 | ρ=0.326, q=0.493 | ρ=-0.283, q=0.528 | ρ=-0.250, q=0.558 | ρ=0.019, q=0.957 | ρ=0.223, q=0.601 | ρ=0.064, q=0.875 | ρ=0.105, q=0.796 | ρ=0.188, q=0.646 |
| Left Accumbens | ρ=-0.213, q=0.609 | ρ=-0.288, q=0.516 | ρ=0.336, q=0.493 | ρ=-0.160, q=0.699 | ρ=-0.244, q=0.562 | ρ=0.108, q=0.793 | ρ=0.061, q=0.882 | ρ=-0.029, q=0.953 | ρ=0.025, q=0.953 | ρ=0.028, q=0.953 |
| Right Amygdala | ρ=-0.530, q=0.179 | ρ=-0.107, q=0.796 | ρ=0.188, q=0.646 | ρ=-0.237, q=0.572 | ρ=0.128, q=0.765 | ρ=0.242, q=0.567 | ρ=0.415, q=0.353 | ρ=0.443, q=0.319 | ρ=0.159, q=0.699 | ρ=0.392, q=0.375 |
| Left Amygdala | ρ=-0.475, q=0.315 | ρ=-0.113, q=0.793 | ρ=0.262, q=0.558 | ρ=-0.249, q=0.558 | ρ=0.101, q=0.796 | ρ=0.277, q=0.528 | ρ=0.277, q=0.528 | ρ=0.367, q=0.436 | ρ=0.016, q=0.964 | ρ=0.258, q=0.558 |
| Brain Stem | ρ=0.140, q=0.731 | ρ=0.232, q=0.575 | ρ=0.254, q=0.558 | ρ=0.123, q=0.770 | ρ=0.164, q=0.699 | ρ=-0.112, q=0.793 | ρ=-0.605, q=0.149 | ρ=-0.519, q=0.179 | ρ=-0.385, q=0.382 | ρ=-0.563, q=0.149 |
| Right Caudate | ρ=-0.303, q=0.511 | ρ=-0.327, q=0.493 | ρ=0.213, q=0.609 | ρ=-0.405, q=0.354 | ρ=-0.150, q=0.711 | ρ=0.187, q=0.646 | ρ=0.252, q=0.558 | ρ=0.231, q=0.575 | ρ=0.015, q=0.964 | ρ=0.240, q=0.567 |
| Left Caudate | ρ=-0.267, q=0.557 | ρ=-0.353, q=0.486 | ρ=0.246, q=0.562 | ρ=-0.409, q=0.353 | ρ=-0.179, q=0.661 | ρ=0.162, q=0.699 | ρ=0.101, q=0.796 | ρ=0.122, q=0.770 | ρ=-0.085, q=0.826 | ρ=0.099, q=0.797 |
| Right Hippocampus | ρ=-0.448, q=0.319 | ρ=-0.214, q=0.609 | ρ=0.321, q=0.499 | ρ=-0.328, q=0.493 | ρ=0.022, q=0.953 | ρ=0.089, q=0.823 | ρ=0.299, q=0.513 | ρ=0.329, q=0.493 | ρ=0.058, q=0.882 | ρ=0.307, q=0.511 |
| Left Hippocampus | ρ=-0.439, q=0.319 | ρ=-0.150, q=0.711 | ρ=0.194, q=0.646 | ρ=-0.305, q=0.511 | ρ=0.087, q=0.826 | ρ=0.163, q=0.699 | ρ=0.280, q=0.528 | ρ=0.327, q=0.493 | ρ=-0.007, q=0.980 | ρ=0.288, q=0.516 |
| Right Inferior Lateral Ventricle | ρ=0.023, q=0.953 | ρ=0.328, q=0.493 | ρ=0.130, q=0.760 | ρ=0.461, q=0.315 | ρ=0.215, q=0.609 | ρ=0.031, q=0.953 | ρ=-0.207, q=0.618 | ρ=-0.206, q=0.618 | ρ=-0.212, q=0.609 | ρ=-0.141, q=0.731 |
| Left Inferior Lateral Ventricle | ρ=-0.127, q=0.767 | ρ=0.171, q=0.678 | ρ=-0.044, q=0.921 | ρ=0.247, q=0.562 | ρ=0.076, q=0.846 | ρ=0.077, q=0.846 | ρ=-0.100, q=0.796 | ρ=-0.177, q=0.663 | ρ=-0.307, q=0.511 | ρ=0.004, q=0.987 |
| Right Lateral Ventricle | ρ=-0.195, q=0.646 | ρ=0.095, q=0.808 | ρ=0.021, q=0.954 | ρ=0.029, q=0.953 | ρ=0.180, q=0.661 | ρ=0.110, q=0.793 | ρ=-0.150, q=0.711 | ρ=-0.075, q=0.846 | ρ=-0.251, q=0.558 | ρ=-0.085, q=0.826 |
| Left Lateral Ventricle | ρ=-0.231, q=0.575 | ρ=0.101, q=0.796 | ρ=0.053, q=0.897 | ρ=0.068, q=0.867 | ρ=0.111, q=0.793 | ρ=0.102, q=0.796 | ρ=-0.158, q=0.700 | ρ=-0.140, q=0.731 | ρ=-0.234, q=0.575 | ρ=-0.080, q=0.843 |
| Right Pallidum | ρ=-0.032, q=0.953 | ρ=-0.270, q=0.557 | ρ=-0.008, q=0.979 | ρ=-0.028, q=0.953 | ρ=-0.259, q=0.558 | ρ=0.296, q=0.513 | ρ=0.109, q=0.793 | ρ=0.025, q=0.953 | ρ=0.294, q=0.513 | ρ=-0.023, q=0.953 |
| Left Pallidum | ρ=0.059, q=0.882 | ρ=-0.085, q=0.826 | ρ=0.117, q=0.791 | ρ=0.114, q=0.793 | ρ=-0.134, q=0.758 | ρ=0.191, q=0.646 | ρ=-0.199, q=0.646 | ρ=-0.188, q=0.646 | ρ=0.033, q=0.953 | ρ=-0.302, q=0.511 |
| Right Putamen | ρ=-0.294, q=0.513 | ρ=-0.457, q=0.315 | ρ=0.198, q=0.646 | ρ=-0.396, q=0.372 | ρ=-0.314, q=0.511 | ρ=0.143, q=0.731 | ρ=0.188, q=0.646 | ρ=0.130, q=0.760 | ρ=0.068, q=0.867 | ρ=0.151, q=0.711 |
| Left Putamen | ρ=-0.339, q=0.493 | ρ=-0.455, q=0.315 | ρ=0.232, q=0.575 | ρ=-0.376, q=0.398 | ρ=-0.308, q=0.511 | ρ=0.194, q=0.646 | ρ=0.176, q=0.663 | ρ=0.161, q=0.699 | ρ=0.059, q=0.882 | ρ=0.125, q=0.768 |
| Right Thalamus | ρ=-0.186, q=0.646 | ρ=-0.363, q=0.440 | ρ=-0.094, q=0.808 | ρ=-0.181, q=0.661 | ρ=-0.311, q=0.511 | ρ=0.170, q=0.681 | ρ=0.125, q=0.768 | ρ=-0.047, q=0.917 | ρ=0.258, q=0.558 | ρ=0.001, q=0.997 |
| Left Thalamus | ρ=-0.149, q=0.711 | ρ=-0.424, q=0.353 | ρ=-0.147, q=0.715 | ρ=-0.152, q=0.711 | ρ=-0.409, q=0.353 | ρ=0.216, q=0.609 | ρ=0.067, q=0.867 | ρ=-0.109, q=0.793 | ρ=0.210, q=0.609 | ρ=-0.074, q=0.850 |
| Right Ventral Diencephalon | ρ=-0.104, q=0.796 | ρ=0.211, q=0.609 | ρ=0.278, q=0.528 | ρ=0.033, q=0.953 | ρ=0.252, q=0.558 | ρ=0.078, q=0.846 | ρ=-0.456, q=0.315 | ρ=-0.349, q=0.492 | ρ=-0.332, q=0.493 | ρ=-0.415, q=0.353 |
| Left Ventral Diencephalon | ρ=-0.131, q=0.760 | ρ=0.162, q=0.699 | ρ=0.252, q=0.558 | ρ=0.058, q=0.882 | ρ=0.185, q=0.646 | ρ=0.044, q=0.921 | ρ=-0.415, q=0.353 | ρ=-0.324, q=0.497 | ρ=-0.280, q=0.528 | ρ=-0.396, q=0.372 |

*FDR corrected p-values <0.05. CPZ: Chlorpromazine equivalent, T-PANSS: Positive and Negative Symptom Scale total score, P-PANSS: Positive and Negative Symptom Scale positive subscale score, N-PANSS: Positive and Negative Symptom Scale negative subscale score, G-PANSS: Positive and Negative Symptom Scale general subscale score.

Supplementary Table 4. Correlation between demographic and clinical characteristics and mean subcortical volumes in ultra treatment-resistant group

| Region of Interest | Sex | Age | Education | Age of Onset | Duration of Illness | CPZ Daily Dose | T-PANSS | P-PANSS | N-PANSS | G-PANSS |
| --- | --- | --- | --- | --- | --- | --- | --- | --- | --- | --- |
| Third Ventricle | ρ=-0.427, q=0.565 | ρ=0.353, q=0.632 | ρ=0.232, q=0.766 | ρ=0.309, q=0.632 | ρ=0.210, q=0.802 | ρ=-0.145, q=0.842 | ρ=-0.388, q=0.609 | ρ=-0.118, q=0.880 | ρ=-0.494, q=0.564 | ρ=-0.287, q=0.662 |
| Fourth Ventricle | ρ=-0.019, q=0.976 | ρ=-0.345, q=0.632 | ρ=0.213, q=0.795 | ρ=-0.044, q=0.960 | ρ=-0.465, q=0.564 | ρ=0.177, q=0.825 | ρ=-0.016, q=0.978 | ρ=0.036, q=0.973 | ρ=0.026, q=0.976 | ρ=0.145, q=0.842 |
| Right Accumbens | ρ=-0.483, q=0.564 | ρ=-0.158, q=0.825 | ρ=0.276, q=0.662 | ρ=0.005, q=0.988 | ρ=-0.174, q=0.825 | ρ=0.049, q=0.955 | ρ=-0.221, q=0.791 | ρ=-0.091, q=0.925 | ρ=-0.477, q=0.564 | ρ=-0.066, q=0.935 |
| Left Accumbens | ρ=-0.334, q=0.632 | ρ=-0.275, q=0.662 | ρ=0.205, q=0.808 | ρ=-0.092, q=0.925 | ρ=-0.184, q=0.825 | ρ=0.081, q=0.933 | ρ=-0.006, q=0.987 | ρ=0.096, q=0.922 | ρ=-0.343, q=0.632 | ρ=0.126, q=0.868 |
| Right Amygdala | ρ=-0.465, q=0.564 | ρ=-0.109, q=0.893 | ρ=0.340, q=0.632 | ρ=0.078, q=0.933 | ρ=-0.282, q=0.662 | ρ=-0.158, q=0.825 | ρ=-0.391, q=0.609 | ρ=-0.360, q=0.632 | ρ=-0.214, q=0.795 | ρ=-0.260, q=0.723 |
| Left Amygdala | ρ=-0.390, q=0.609 | ρ=0.038, q=0.971 | ρ=0.416, q=0.565 | ρ=0.161, q=0.825 | ρ=-0.175, q=0.825 | ρ=-0.169, q=0.825 | ρ=-0.323, q=0.632 | ρ=-0.435, q=0.565 | ρ=-0.031, q=0.973 | ρ=-0.233, q=0.766 |
| Brain Stem | ρ=-0.130, q=0.859 | ρ=-0.055, q=0.937 | ρ=-0.103, q=0.902 | ρ=-0.275, q=0.662 | ρ=0.223, q=0.789 | ρ=0.343, q=0.632 | ρ=0.071, q=0.935 | ρ=0.414, q=0.565 | ρ=-0.312, q=0.632 | ρ=0.018, q=0.976 |
| Right Caudate | ρ=-0.334, q=0.632 | ρ=-0.065, q=0.935 | ρ=0.299, q=0.636 | ρ=0.022, q=0.976 | ρ=-0.070, q=0.935 | ρ=-0.161, q=0.825 | ρ=-0.032, q=0.973 | ρ=-0.169, q=0.825 | ρ=-0.275, q=0.662 | ρ=0.195, q=0.820 |
| Left Caudate | ρ=-0.316, q=0.632 | ρ=-0.007, q=0.987 | ρ=0.319, q=0.632 | ρ=0.084, q=0.933 | ρ=-0.057, q=0.937 | ρ=-0.167, q=0.825 | ρ=-0.065, q=0.935 | ρ=-0.193, q=0.820 | ρ=-0.300, q=0.636 | ρ=0.142, q=0.844 |
| Right Hippocampus | ρ=-0.316, q=0.632 | ρ=-0.224, q=0.789 | ρ=0.195, q=0.820 | ρ=0.166, q=0.825 | ρ=-0.472, q=0.564 | ρ=0.025, q=0.976 | ρ=-0.121, q=0.880 | ρ=-0.180, q=0.825 | ρ=-0.064, q=0.935 | ρ=-0.071, q=0.935 |
| Left Hippocampus | ρ=-0.334, q=0.632 | ρ=-0.251, q=0.729 | ρ=0.189, q=0.825 | ρ=0.109, q=0.893 | ρ=-0.409, q=0.565 | ρ=0.035, q=0.973 | ρ=-0.146, q=0.842 | ρ=-0.107, q=0.893 | ρ=-0.124, q=0.871 | ρ=-0.001, q=0.996 |
| Right Inferior Lateral Ventricle | ρ=-0.186, q=0.825 | ρ=0.232, q=0.766 | ρ=0.050, q=0.954 | ρ=0.081, q=0.933 | ρ=0.275, q=0.662 | ρ=0.059, q=0.937 | ρ=-0.022, q=0.976 | ρ=-0.044, q=0.960 | ρ=-0.138, q=0.844 | ρ=-0.172, q=0.825 |
| Left Inferior Lateral Ventricle | ρ=-0.200, q=0.820 | ρ=0.330, q=0.632 | ρ=0.261, q=0.729 | ρ=0.318, q=0.632 | ρ=0.145, q=0.844 | ρ=0.092, q=0.925 | ρ=0.023, q=0.976 | ρ=0.118, q=0.882 | ρ=-0.290, q=0.662 | ρ=-0.082, q=0.933 |
| Right Lateral Ventricle | ρ=-0.446, q=0.565 | ρ=0.175, q=0.825 | ρ=0.424, q=0.565 | ρ=0.089, q=0.925 | ρ=0.176, q=0.825 | ρ=-0.010, q=0.983 | ρ=-0.339, q=0.632 | ρ=-0.162, q=0.825 | ρ=-0.560, q=0.519 | ρ=-0.174, q=0.825 |
| Left Lateral Ventricle | ρ=-0.372, q=0.632 | ρ=0.232, q=0.766 | ρ=0.433, q=0.565 | ρ=0.186, q=0.825 | ρ=0.118, q=0.880 | ρ=-0.020, q=0.976 | ρ=-0.308, q=0.632 | ρ=-0.151, q=0.842 | ρ=-0.569, q=0.519 | ρ=-0.165, q=0.825 |
| Right Pallidum | ρ=-0.409, q=0.565 | ρ=-0.201, q=0.813 | ρ=-0.250, q=0.729 | ρ=-0.165, q=0.825 | ρ=-0.060, q=0.937 | ρ=0.436, q=0.565 | ρ=0.032, q=0.973 | ρ=0.250, q=0.729 | ρ=-0.428, q=0.565 | ρ=0.012, q=0.983 |
| Left Pallidum | ρ=-0.409, q=0.565 | ρ=-0.107, q=0.893 | ρ=-0.069, q=0.935 | ρ=-0.117, q=0.881 | ρ=0.063, q=0.935 | ρ=0.495, q=0.564 | ρ=0.071, q=0.935 | ρ=0.325, q=0.632 | ρ=-0.473, q=0.564 | ρ=0.031, q=0.973 |
| Right Putamen | ρ=-0.279, q=0.662 | ρ=-0.129, q=0.859 | ρ=0.011, q=0.983 | ρ=-0.140, q=0.844 | ρ=-0.019, q=0.976 | ρ=0.323, q=0.632 | ρ=0.157, q=0.825 | ρ=0.162, q=0.825 | ρ=-0.283, q=0.662 | ρ=0.268, q=0.690 |
| Left Putamen | ρ=-0.204, q=0.808 | ρ=-0.129, q=0.859 | ρ=0.067, q=0.935 | ρ=-0.055, q=0.937 | ρ=-0.064, q=0.935 | ρ=0.403, q=0.577 | ρ=0.200, q=0.813 | ρ=0.148, q=0.842 | ρ=-0.186, q=0.825 | ρ=0.213, q=0.795 |
| Right Thalamus | ρ=0.130, q=0.859 | ρ=-0.332, q=0.632 | ρ=0.148, q=0.842 | ρ=-0.251, q=0.729 | ρ=-0.102, q=0.902 | ρ=0.305, q=0.632 | ρ=0.079, q=0.933 | ρ=0.346, q=0.632 | ρ=-0.207, q=0.808 | ρ=-0.041, q=0.964 |
| Left Thalamus | ρ=0.353, q=0.632 | ρ=-0.604, q=0.519 | ρ=-0.137, q=0.844 | ρ=-0.215, q=0.795 | ρ=-0.540, q=0.543 | ρ=0.249, q=0.729 | ρ=0.329, q=0.632 | ρ=0.278, q=0.662 | ρ=0.139, q=0.844 | ρ=0.307, q=0.632 |
| Right Ventral Diencephalon | ρ=-0.223, q=0.789 | ρ=-0.247, q=0.729 | ρ=0.010, q=0.983 | ρ=-0.375, q=0.632 | ρ=0.083, q=0.933 | ρ=0.359, q=0.632 | ρ=0.045, q=0.960 | ρ=0.338, q=0.632 | ρ=-0.242, q=0.746 | ρ=0.018, q=0.976 |
| Left Ventral Diencephalon | ρ=-0.149, q=0.842 | ρ=-0.301, q=0.636 | ρ=0.061, q=0.937 | ρ=-0.342, q=0.632 | ρ=-0.091, q=0.925 | ρ=0.246, q=0.729 | ρ=0.108, q=0.893 | ρ=0.342, q=0.632 | ρ=-0.216, q=0.795 | ρ=0.088, q=0.925 |

*FDR corrected p-values <0.05. CPZ: Chlorpromazine equivalent, T-PANSS: Positive and Negative Symptom Scale total score, P-PANSS: Positive and Negative Symptom Scale positive subscale score, N-PANSS: Positive and Negative Symptom Scale negative subscale score, G-PANSS: Positive and Negative Symptom Scale general subscale score.

Supplementary Table 5. Partial correlation controlling for chlorpromazine-equivalent daily dose between demographic and clinical characteristics and mean subcortical volumes in all participants

| Region of Interest | Sex | Age | Education | Age of Onset | Duration of Illness | T-PANSS | P-PANSS | N-PANSS | G-PANSS |
| --- | --- | --- | --- | --- | --- | --- | --- | --- | --- |
| Third Ventricle | ρ=-0.345, q=0.055 | ρ=0.328, q=0.055 | ρ=0.131, q=0.564 | ρ=0.121, q=0.593 | ρ=0.260, q=0.194 | ρ=-0.001, q=0.996 | ρ=-0.038, q=0.939 | ρ=-0.062, q=0.895 | ρ=0.028, q=0.945 |
| Fourth Ventricle | ρ=-0.040, q=0.929 | ρ=-0.329, q=0.055 | ρ=0.170, q=0.444 | ρ=-0.185, q=0.394 | ρ=-0.260, q=0.194 | ρ=0.042, q=0.929 | ρ=-0.016, q=0.961 | ρ=0.019, q=0.958 | ρ=0.120, q=0.593 |
| Right Accumbens | ρ=-0.346, q=0.055 | ρ=-0.184, q=0.394 | ρ=0.240, q=0.239 | ρ=-0.207, q=0.322 | ρ=-0.068, q=0.877 | ρ=0.042, q=0.929 | ρ=-0.002, q=0.996 | ρ=-0.004, q=0.996 | ρ=0.024, q=0.951 |
| Left Accumbens | ρ=-0.331, q=0.055 | ρ=-0.167, q=0.452 | ρ=0.235, q=0.243 | ρ=-0.193, q=0.394 | ρ=-0.055, q=0.911 | ρ=0.013, q=0.970 | ρ=-0.022, q=0.951 | ρ=0.010, q=0.979 | ρ=-0.034, q=0.944 |
| Right Amygdala | ρ=-0.421*, q=0.007 | ρ=-0.218, q=0.276 | ρ=0.141, q=0.530 | ρ=-0.166, q=0.452 | ρ=-0.048, q=0.929 | ρ=0.142, q=0.529 | ρ=0.072, q=0.864 | ρ=0.077, q=0.862 | ρ=0.140, q=0.530 |
| Left Amygdala | ρ=-0.420*, q=0.007 | ρ=-0.171, q=0.444 | ρ=0.188, q=0.394 | ρ=-0.184, q=0.394 | ρ=0.001, q=0.996 | ρ=0.066, q=0.890 | ρ=-0.001, q=0.996 | ρ=0.008, q=0.986 | ρ=0.059, q=0.905 |
| Brain Stem | ρ=0.018, q=0.959 | ρ=0.184, q=0.394 | ρ=0.128, q=0.570 | ρ=-0.001, q=0.996 | ρ=0.136, q=0.550 | ρ=-0.329, q=0.055 | ρ=-0.063, q=0.893 | ρ=-0.281, q=0.144 | ρ=-0.325, q=0.055 |
| Right Caudate | ρ=-0.247, q=0.211 | ρ=-0.257, q=0.194 | ρ=0.183, q=0.394 | ρ=-0.231, q=0.249 | ρ=-0.095, q=0.742 | ρ=0.063, q=0.893 | ρ=0.054, q=0.911 | ρ=-0.025, q=0.951 | ρ=0.064, q=0.893 |
| Left Caudate | ρ=-0.232, q=0.247 | ρ=-0.288, q=0.138 | ρ=0.221, q=0.276 | ρ=-0.255, q=0.194 | ρ=-0.120, q=0.593 | ρ=0.031, q=0.944 | ρ=0.017, q=0.961 | ρ=-0.043, q=0.929 | ρ=0.024, q=0.951 |
| Right Hippocampus | ρ=-0.419*, q=0.007 | ρ=-0.136, q=0.550 | ρ=0.146, q=0.520 | ρ=-0.158, q=0.477 | ρ=-0.025, q=0.951 | ρ=0.129, q=0.570 | ρ=0.053, q=0.911 | ρ=0.063, q=0.893 | ρ=0.135, q=0.551 |
| Left Hippocampus | ρ=-0.468*, q=0.003 | ρ=-0.153, q=0.492 | ρ=0.154, q=0.492 | ρ=-0.161, q=0.467 | ρ=-0.049, q=0.929 | ρ=0.102, q=0.700 | ρ=0.102, q=0.700 | ρ=-0.001, q=0.996 | ρ=0.121, q=0.593 |
| Right Inferior Lateral Ventricle | ρ=-0.152, q=0.492 | ρ=0.128, q=0.570 | ρ=0.068, q=0.877 | ρ=0.133, q=0.557 | ρ=0.150, q=0.492 | ρ=-0.140, q=0.530 | ρ=-0.186, q=0.394 | ρ=-0.112, q=0.647 | ρ=-0.111, q=0.647 |
| Left Inferior Lateral Ventricle | ρ=-0.272, q=0.175 | ρ=0.100, q=0.717 | ρ=0.040, q=0.929 | ρ=0.166, q=0.456 | ρ=0.003, q=0.996 | ρ=0.053, q=0.911 | ρ=-0.022, q=0.951 | ρ=-0.014, q=0.970 | ρ=0.071, q=0.877 |
| Right Lateral Ventricle | ρ=-0.227, q=0.262 | ρ=0.076, q=0.864 | ρ=0.183, q=0.394 | ρ=0.023, q=0.951 | ρ=0.043, q=0.929 | ρ=-0.060, q=0.903 | ρ=-0.079, q=0.853 | ρ=-0.126, q=0.575 | ρ=-0.034, q=0.944 |
| Left Lateral Ventricle | ρ=-0.183, q=0.394 | ρ=0.073, q=0.864 | ρ=0.152, q=0.492 | ρ=0.033, q=0.944 | ρ=0.023, q=0.951 | ρ=-0.012, q=0.972 | ρ=-0.103, q=0.700 | ρ=-0.069, q=0.877 | ρ=-0.004, q=0.996 |
| Right Pallidum | ρ=-0.054, q=0.911 | ρ=-0.102, q=0.700 | ρ=0.074, q=0.864 | ρ=0.035, q=0.944 | ρ=-0.176, q=0.422 | ρ=-0.215, q=0.285 | ρ=-0.021, q=0.954 | ρ=-0.226, q=0.262 | ρ=-0.176, q=0.422 |
| Left Pallidum | ρ=-0.031, q=0.944 | ρ=0.041, q=0.929 | ρ=0.073, q=0.864 | ρ=0.142, q=0.529 | ρ=-0.126, q=0.575 | ρ=-0.161, q=0.467 | ρ=0.049, q=0.929 | ρ=-0.151, q=0.492 | ρ=-0.166, q=0.452 |
| Right Putamen | ρ=-0.219, q=0.276 | ρ=-0.255, q=0.194 | ρ=0.144, q=0.526 | ρ=-0.187, q=0.394 | ρ=-0.154, q=0.492 | ρ=0.020, q=0.955 | ρ=0.031, q=0.944 | ρ=-0.046, q=0.929 | ρ=0.047, q=0.929 |
| Left Putamen | ρ=-0.217, q=0.276 | ρ=-0.235, q=0.243 | ρ=0.171, q=0.444 | ρ=-0.164, q=0.456 | ρ=-0.132, q=0.561 | ρ=0.057, q=0.911 | ρ=0.031, q=0.944 | ρ=0.009, q=0.986 | ρ=0.046, q=0.929 |
| Right Thalamus | ρ=-0.151, q=0.492 | ρ=-0.220, q=0.276 | ρ=-0.054, q=0.911 | ρ=-0.252, q=0.195 | ρ=-0.041, q=0.929 | ρ=-0.013, q=0.970 | ρ=-0.031, q=0.944 | ρ=0.028, q=0.945 | ρ=-0.093, q=0.753 |
| Left Thalamus | ρ=-0.075, q=0.864 | ρ=-0.341, q=0.055 | ρ=-0.117, q=0.612 | ρ=-0.252, q=0.195 | ρ=-0.182, q=0.394 | ρ=-0.041, q=0.929 | ρ=-0.083, q=0.818 | ρ=0.028, q=0.945 | ρ=-0.089, q=0.765 |
| Right Ventral Diencephalon | ρ=-0.187, q=0.394 | ρ=0.096, q=0.742 | ρ=0.236, q=0.243 | ρ=-0.042, q=0.929 | ρ=0.091, q=0.761 | ρ=-0.325, q=0.055 | ρ=-0.090, q=0.762 | ρ=-0.326, q=0.055 | ρ=-0.294, q=0.125 |
| Left Ventral Diencephalon | ρ=-0.206, q=0.322 | ρ=0.122, q=0.593 | ρ=0.180, q=0.403 | ρ=-0.030, q=0.944 | ρ=0.102, q=0.700 | ρ=-0.279, q=0.144 | ρ=-0.053, q=0.911 | ρ=-0.283, q=0.144 | ρ=-0.256, q=0.194 |

*FDR corrected p-values <0.05. T-PANSS: Positive and Negative Symptom Scale total score, P-PANSS: Positive and Negative Symptom Scale positive subscale score, N-PANSS: Positive and Negative Symptom Scale negative subscale score, G-PANSS: Positive and Negative Symptom Scale general subscale score.

Supplementary Table 6. Partial correlation controlling for chlorpromazine-equivalent daily dose between demographic and clinical characteristics and mean subcortical volumes in first-line responders

| Region of Interest | Sex | Age | Education | Age of Onset | Duration of Illness | T-PANSS | P-PANSS | N-PANSS | G-PANSS |
| --- | --- | --- | --- | --- | --- | --- | --- | --- | --- |
| Third Ventricle | ρ=-0.170, q=0.909 | ρ=0.549, q=0.256 | ρ=-0.044, q=0.985 | ρ=0.275, q=0.749 | ρ=0.108, q=0.985 | ρ=0.051, q=0.985 | ρ=-0.149, q=0.934 | ρ=0.001, q=0.995 | ρ=0.009, q=0.985 |
| Fourth Ventricle | ρ=0.086, q=0.985 | ρ=-0.277, q=0.749 | ρ=0.140, q=0.935 | ρ=-0.122, q=0.985 | ρ=-0.352, q=0.593 | ρ=0.044, q=0.985 | ρ=-0.229, q=0.822 | ρ=-0.052, q=0.985 | ρ=0.184, q=0.856 |
| Right Accumbens | ρ=-0.354, q=0.593 | ρ=-0.166, q=0.909 | ρ=0.095, q=0.985 | ρ=-0.384, q=0.593 | ρ=0.156, q=0.934 | ρ=-0.204, q=0.837 | ρ=0.011, q=0.985 | ρ=-0.298, q=0.713 | ρ=-0.275, q=0.749 |
| Left Accumbens | ρ=-0.416, q=0.593 | ρ=-0.041, q=0.985 | ρ=0.050, q=0.985 | ρ=-0.330, q=0.644 | ρ=0.211, q=0.822 | ρ=-0.191, q=0.837 | ρ=-0.099, q=0.985 | ρ=-0.198, q=0.837 | ρ=-0.331, q=0.644 |
| Right Amygdala | ρ=-0.372, q=0.593 | ρ=-0.355, q=0.593 | ρ=-0.099, q=0.985 | ρ=-0.247, q=0.816 | ρ=-0.060, q=0.985 | ρ=0.048, q=0.985 | ρ=0.083, q=0.985 | ρ=0.024, q=0.985 | ρ=-0.014, q=0.985 |
| Left Amygdala | ρ=-0.449, q=0.513 | ρ=-0.329, q=0.644 | ρ=-0.104, q=0.985 | ρ=-0.412, q=0.593 | ρ=0.068, q=0.985 | ρ=0.045, q=0.985 | ρ=0.052, q=0.985 | ρ=0.021, q=0.985 | ρ=-0.025, q=0.985 |
| Brain Stem | ρ=-0.060, q=0.985 | ρ=0.273, q=0.749 | ρ=0.150, q=0.934 | ρ=0.082, q=0.985 | ρ=0.098, q=0.985 | ρ=-0.229, q=0.822 | ρ=0.148, q=0.934 | ρ=-0.237, q=0.822 | ρ=-0.192, q=0.837 |
| Right Caudate | ρ=-0.216, q=0.822 | ρ=-0.311, q=0.678 | ρ=-0.123, q=0.985 | ρ=-0.377, q=0.593 | ρ=0.056, q=0.985 | ρ=-0.140, q=0.935 | ρ=0.007, q=0.985 | ρ=-0.231, q=0.822 | ρ=-0.248, q=0.816 |
| Left Caudate | ρ=-0.233, q=0.822 | ρ=-0.373, q=0.593 | ρ=-0.063, q=0.985 | ρ=-0.438, q=0.513 | ρ=0.028, q=0.985 | ρ=-0.098, q=0.985 | ρ=0.029, q=0.985 | ρ=-0.221, q=0.822 | ρ=-0.215, q=0.822 |
| Right Hippocampus | ρ=-0.465, q=0.513 | ρ=-0.008, q=0.985 | ρ=-0.114, q=0.985 | ρ=-0.283, q=0.749 | ρ=0.261, q=0.794 | ρ=0.014, q=0.985 | ρ=-0.017, q=0.985 | ρ=0.095, q=0.985 | ρ=-0.140, q=0.935 |
| Left Hippocampus | ρ=-0.588, q=0.206 | ρ=-0.068, q=0.985 | ρ=-0.010, q=0.985 | ρ=-0.271, q=0.752 | ρ=0.150, q=0.934 | ρ=0.153, q=0.934 | ρ=0.129, q=0.980 | ρ=0.097, q=0.985 | ρ=-0.005, q=0.990 |
| Right Inferior Lateral Ventricle | ρ=-0.510, q=0.384 | ρ=-0.213, q=0.822 | ρ=0.056, q=0.985 | ρ=0.055, q=0.985 | ρ=-0.190, q=0.837 | ρ=-0.214, q=0.822 | ρ=-0.218, q=0.822 | ρ=-0.073, q=0.985 | ρ=-0.097, q=0.985 |
| Left Inferior Lateral Ventricle | ρ=-0.457, q=0.513 | ρ=-0.062, q=0.985 | ρ=-0.045, q=0.985 | ρ=0.140, q=0.935 | ρ=-0.276, q=0.749 | ρ=-0.064, q=0.985 | ρ=-0.106, q=0.985 | ρ=-0.077, q=0.985 | ρ=-0.090, q=0.985 |
| Right Lateral Ventricle | ρ=-0.189, q=0.837 | ρ=0.040, q=0.985 | ρ=0.090, q=0.985 | ρ=0.108, q=0.985 | ρ=-0.195, q=0.837 | ρ=0.101, q=0.985 | ρ=0.043, q=0.985 | ρ=-0.166, q=0.909 | ρ=0.114, q=0.985 |
| Left Lateral Ventricle | ρ=-0.063, q=0.985 | ρ=-0.024, q=0.985 | ρ=-0.086, q=0.985 | ρ=0.066, q=0.985 | ρ=-0.176, q=0.892 | ρ=0.084, q=0.985 | ρ=-0.043, q=0.985 | ρ=-0.147, q=0.934 | ρ=0.027, q=0.985 |
| Right Pallidum | ρ=-0.199, q=0.837 | ρ=0.123, q=0.985 | ρ=0.309, q=0.678 | ρ=0.197, q=0.837 | ρ=-0.145, q=0.935 | ρ=-0.069, q=0.985 | ρ=0.228, q=0.822 | ρ=-0.367, q=0.593 | ρ=-0.012, q=0.985 |
| Left Pallidum | ρ=-0.094, q=0.985 | ρ=0.289, q=0.749 | ρ=0.083, q=0.985 | ρ=0.320, q=0.660 | ρ=-0.147, q=0.934 | ρ=0.076, q=0.985 | ρ=0.321, q=0.660 | ρ=-0.095, q=0.985 | ρ=0.022, q=0.985 |
| Right Putamen | ρ=-0.243, q=0.816 | ρ=-0.246, q=0.816 | ρ=-0.002, q=0.995 | ρ=-0.258, q=0.798 | ρ=-0.010, q=0.985 | ρ=0.021, q=0.985 | ρ=0.014, q=0.985 | ρ=-0.090, q=0.985 | ρ=-0.072, q=0.985 |
| Left Putamen | ρ=-0.245, q=0.816 | ρ=-0.179, q=0.883 | ρ=-0.036, q=0.985 | ρ=-0.263, q=0.794 | ρ=0.068, q=0.985 | ρ=0.041, q=0.985 | ρ=-0.028, q=0.985 | ρ=-0.009, q=0.985 | ρ=-0.090, q=0.985 |
| Right Thalamus | ρ=-0.342, q=0.623 | ρ=0.053, q=0.985 | ρ=-0.213, q=0.822 | ρ=-0.344, q=0.623 | ρ=0.395, q=0.593 | ρ=-0.168, q=0.909 | ρ=-0.156, q=0.934 | ρ=-0.082, q=0.985 | ρ=-0.318, q=0.660 |
| Left Thalamus | ρ=-0.247, q=0.816 | ρ=-0.037, q=0.985 | ρ=-0.121, q=0.985 | ρ=-0.438, q=0.513 | ρ=0.401, q=0.593 | ρ=-0.190, q=0.837 | ρ=-0.158, q=0.934 | ρ=-0.087, q=0.985 | ρ=-0.307, q=0.678 |
| Right Ventral Diencephalon | ρ=-0.404, q=0.593 | ρ=0.215, q=0.822 | ρ=0.356, q=0.593 | ρ=0.032, q=0.985 | ρ=0.068, q=0.985 | ρ=-0.130, q=0.980 | ρ=0.197, q=0.837 | ρ=-0.305, q=0.678 | ρ=-0.034, q=0.985 |
| Left Ventral Diencephalon | ρ=-0.381, q=0.593 | ρ=0.287, q=0.749 | ρ=0.215, q=0.822 | ρ=0.026, q=0.985 | ρ=0.168, q=0.909 | ρ=-0.052, q=0.985 | ρ=0.197, q=0.837 | ρ=-0.211, q=0.822 | ρ=-0.017, q=0.985 |

*FDR corrected p-values <0.05. T-PANSS: Positive and Negative Symptom Scale total score, P-PANSS: Positive and Negative Symptom Scale positive subscale score, N-PANSS: Positive and Negative Symptom Scale negative subscale score, G-PANSS: Positive and Negative Symptom Scale general subscale score.

Supplementary Table 7. Partial correlation controlling for chlorpromazine-equivalent daily dose between demographic and clinical characteristics and mean subcortical volumes in clozapine responders

| Region of Interest | Sex | Age | Education | Age of Onset | Duration of Illness | T-PANSS | P-PANSS | N-PANSS | G-PANSS |
| --- | --- | --- | --- | --- | --- | --- | --- | --- | --- |
| Third Ventricle | ρ=-0.483, q=0.258 | ρ=0.248, q=0.534 | ρ=0.250, q=0.534 | ρ=0.003, q=0.992 | ρ=0.354, q=0.411 | ρ=-0.082, q=0.851 | ρ=0.135, q=0.752 | ρ=-0.323, q=0.428 | ρ=0.065, q=0.869 |
| Fourth Ventricle | ρ=-0.104, q=0.812 | ρ=-0.340, q=0.411 | ρ=0.116, q=0.799 | ρ=-0.420, q=0.311 | ρ=-0.067, q=0.869 | ρ=0.213, q=0.575 | ρ=0.499, q=0.258 | ρ=0.154, q=0.707 | ρ=0.187, q=0.638 |
| Right Accumbens | ρ=-0.276, q=0.515 | ρ=-0.343, q=0.411 | ρ=0.336, q=0.411 | ρ=-0.284, q=0.496 | ρ=-0.257, q=0.534 | ρ=0.225, q=0.568 | ρ=0.062, q=0.869 | ρ=0.103, q=0.812 | ρ=0.187, q=0.638 |
| Left Accumbens | ρ=-0.184, q=0.644 | ρ=-0.302, q=0.490 | ρ=0.365, q=0.395 | ρ=-0.166, q=0.683 | ρ=-0.268, q=0.530 | ρ=0.037, q=0.915 | ρ=-0.081, q=0.851 | ρ=0.015, q=0.968 | ρ=0.013, q=0.972 |
| Right Amygdala | ρ=-0.486, q=0.258 | ρ=-0.135, q=0.752 | ρ=0.245, q=0.534 | ρ=-0.256, q=0.534 | ρ=0.092, q=0.825 | ρ=0.382, q=0.367 | ρ=0.389, q=0.366 | ρ=0.143, q=0.744 | ρ=0.373, q=0.367 |
| Left Amygdala | ρ=-0.405, q=0.324 | ρ=-0.146, q=0.732 | ρ=0.333, q=0.411 | ρ=-0.272, q=0.517 | ρ=0.058, q=0.877 | ρ=0.229, q=0.559 | ρ=0.289, q=0.496 | ρ=-0.008, q=0.985 | ρ=0.231, q=0.559 |
| Brain Stem | ρ=0.098, q=0.820 | ρ=0.245, q=0.534 | ρ=0.239, q=0.543 | ρ=0.129, q=0.761 | ρ=0.187, q=0.638 | ρ=-0.599, q=0.201 | ρ=-0.522, q=0.258 | ρ=-0.379, q=0.367 | ρ=-0.557, q=0.258 |
| Right Caudate | ρ=-0.247, q=0.534 | ρ=-0.353, q=0.411 | ρ=0.257, q=0.534 | ρ=-0.421, q=0.311 | ρ=-0.187, q=0.638 | ρ=0.219, q=0.568 | ρ=0.172, q=0.678 | ρ=-0.001, q=0.995 | ρ=0.220, q=0.568 |
| Left Caudate | ρ=-0.218, q=0.568 | ρ=-0.375, q=0.367 | ρ=0.285, q=0.496 | ρ=-0.422, q=0.311 | ρ=-0.212, q=0.575 | ρ=0.067, q=0.869 | ρ=0.062, q=0.869 | ρ=-0.101, q=0.812 | ρ=0.079, q=0.854 |
| Right Hippocampus | ρ=-0.466, q=0.258 | ρ=-0.224, q=0.568 | ρ=0.346, q=0.411 | ρ=-0.334, q=0.411 | ρ=0.007, q=0.985 | ρ=0.287, q=0.496 | ρ=0.322, q=0.428 | ρ=0.050, q=0.890 | ρ=0.299, q=0.490 |
| Left Hippocampus | ρ=-0.418, q=0.311 | ρ=-0.169, q=0.683 | ρ=0.232, q=0.559 | ρ=-0.317, q=0.439 | ρ=0.062, q=0.869 | ρ=0.253, q=0.534 | ρ=0.289, q=0.496 | ρ=-0.022, q=0.956 | ρ=0.272, q=0.517 |
| Right Inferior Lateral Ventricle | ρ=0.044, q=0.898 | ρ=0.326, q=0.425 | ρ=0.139, q=0.750 | ρ=0.460, q=0.258 | ρ=0.213, q=0.575 | ρ=-0.220, q=0.568 | ρ=-0.240, q=0.543 | ρ=-0.215, q=0.575 | ρ=-0.147, q=0.732 |
| Left Inferior Lateral Ventricle | ρ=-0.103, q=0.812 | ρ=0.165, q=0.683 | ρ=-0.030, q=0.939 | ρ=0.244, q=0.534 | ρ=0.064, q=0.869 | ρ=-0.121, q=0.788 | ρ=-0.229, q=0.559 | ρ=-0.315, q=0.439 | ρ=-0.006, q=0.985 |
| Right Lateral Ventricle | ρ=-0.162, q=0.685 | ρ=0.085, q=0.845 | ρ=0.042, q=0.898 | ρ=0.025, q=0.953 | ρ=0.164, q=0.683 | ρ=-0.181, q=0.650 | ρ=-0.133, q=0.752 | ρ=-0.263, q=0.534 | ρ=-0.102, q=0.812 |
| Left Lateral Ventricle | ρ=-0.209, q=0.585 | ρ=0.092, q=0.825 | ρ=0.074, q=0.863 | ρ=0.064, q=0.869 | ρ=0.095, q=0.823 | ρ=-0.187, q=0.638 | ρ=-0.201, q=0.611 | ρ=-0.245, q=0.534 | ρ=-0.095, q=0.823 |
| Right Pallidum | ρ=0.138, q=0.750 | ρ=-0.314, q=0.439 | ρ=0.050, q=0.890 | ρ=-0.043, q=0.898 | ρ=-0.327, q=0.425 | ρ=0.045, q=0.898 | ρ=-0.110, q=0.812 | ρ=0.282, q=0.501 | ρ=-0.067, q=0.869 |
| Left Pallidum | ρ=0.179, q=0.651 | ρ=-0.106, q=0.812 | ρ=0.158, q=0.690 | ρ=0.108, q=0.812 | ρ=-0.171, q=0.678 | ρ=-0.254, q=0.534 | ρ=-0.297, q=0.490 | ρ=0.017, q=0.966 | ρ=-0.338, q=0.411 |
| Right Putamen | ρ=-0.260, q=0.534 | ρ=-0.478, q=0.258 | ρ=0.232, q=0.559 | ρ=-0.406, q=0.324 | ρ=-0.346, q=0.411 | ρ=0.162, q=0.685 | ρ=0.080, q=0.854 | ρ=0.056, q=0.877 | ρ=0.134, q=0.752 |
| Left Putamen | ρ=-0.286, q=0.496 | ρ=-0.485, q=0.258 | ρ=0.279, q=0.505 | ρ=-0.392, q=0.366 | ρ=-0.352, q=0.411 | ρ=0.139, q=0.750 | ρ=0.091, q=0.825 | ρ=0.043, q=0.898 | ρ=0.101, q=0.812 |
| Right Thalamus | ρ=-0.119, q=0.793 | ρ=-0.387, q=0.366 | ρ=-0.064, q=0.869 | ρ=-0.191, q=0.638 | ρ=-0.350, q=0.411 | ρ=0.090, q=0.825 | ρ=-0.129, q=0.761 | ρ=0.248, q=0.534 | ρ=-0.023, q=0.956 |
| Left Thalamus | ρ=-0.050, q=0.890 | ρ=-0.458, q=0.258 | ρ=-0.111, q=0.812 | ρ=-0.165, q=0.683 | ρ=-0.463, q=0.258 | ρ=0.019, q=0.963 | ρ=-0.222, q=0.568 | ρ=0.197, q=0.624 | ρ=-0.107, q=0.812 |
| Right Ventral Diencephalon | ρ=-0.076, q=0.861 | ρ=0.205, q=0.594 | ρ=0.298, q=0.490 | ρ=0.029, q=0.939 | ρ=0.243, q=0.536 | ρ=-0.488, q=0.258 | ρ=-0.419, q=0.311 | ρ=-0.341, q=0.411 | ρ=-0.431, q=0.311 |
| Left Ventral Diencephalon | ρ=-0.126, q=0.769 | ρ=0.159, q=0.690 | ρ=0.265, q=0.534 | ρ=0.056, q=0.877 | ρ=0.181, q=0.650 | ρ=-0.437, q=0.311 | ρ=-0.375, q=0.367 | ρ=-0.285, q=0.496 | ρ=-0.406, q=0.324 |

*FDR corrected p-values <0.05. T-PANSS: Positive and Negative Symptom Scale total score, P-PANSS: Positive and Negative Symptom Scale positive subscale score, N-PANSS: Positive and Negative Symptom Scale negative subscale score, G-PANSS: Positive and Negative Symptom Scale general subscale score.

Supplementary Table 8. Partial correlation controlling for chlorpromazine-equivalent daily dose between demographic and clinical characteristics and mean subcortical volumes in ultra treatment-resistant group

| Region of Interest | Sex | Age | Education | Age of Onset | Duration of Illness | T-PANSS | P-PANSS | N-PANSS | G-PANSS |
| --- | --- | --- | --- | --- | --- | --- | --- | --- | --- |
| Third Ventricle | ρ=-0.435, q=0.563 | ρ=0.353, q=0.755 | ρ=0.203, q=0.822 | ρ=0.316, q=0.755 | ρ=0.224, q=0.814 | ρ=-0.364, q=0.755 | ρ=-0.040, q=0.934 | ρ=-0.498, q=0.520 | ρ=-0.270, q=0.755 |
| Fourth Ventricle | ρ=-0.016, q=0.974 | ρ=-0.346, q=0.755 | ρ=0.271, q=0.755 | ρ=-0.049, q=0.934 | ρ=-0.489, q=0.520 | ρ=-0.090, q=0.911 | ρ=-0.088, q=0.913 | ρ=0.025, q=0.955 | ρ=0.119, q=0.893 |
| Right Accumbens | ρ=-0.483, q=0.520 | ρ=-0.157, q=0.842 | ρ=0.298, q=0.755 | ρ=0.003, q=0.993 | ρ=-0.179, q=0.842 | ρ=-0.258, q=0.787 | ρ=-0.149, q=0.861 | ρ=-0.478, q=0.520 | ρ=-0.075, q=0.930 |
| Left Accumbens | ρ=-0.334, q=0.755 | ρ=-0.274, q=0.755 | ρ=0.235, q=0.814 | ρ=-0.094, q=0.911 | ρ=-0.192, q=0.838 | ρ=-0.040, q=0.934 | ρ=0.060, q=0.934 | ρ=-0.345, q=0.755 | ρ=0.114, q=0.893 |
| Right Amygdala | ρ=-0.474, q=0.520 | ρ=-0.115, q=0.893 | ρ=0.314, q=0.755 | ρ=0.083, q=0.926 | ρ=-0.274, q=0.755 | ρ=-0.362, q=0.755 | ρ=-0.335, q=0.755 | ρ=-0.216, q=0.820 | ρ=-0.241, q=0.814 |
| Left Amygdala | ρ=-0.399, q=0.688 | ρ=0.034, q=0.947 | ρ=0.391, q=0.715 | ρ=0.168, q=0.842 | ρ=-0.164, q=0.842 | ρ=-0.284, q=0.755 | ρ=-0.422, q=0.620 | ρ=-0.030, q=0.951 | ρ=-0.211, q=0.820 |
| Brain Stem | ρ=-0.132, q=0.880 | ρ=-0.049, q=0.934 | ρ=-0.017, q=0.974 | ρ=-0.302, q=0.755 | ρ=0.209, q=0.820 | ρ=-0.067, q=0.932 | ρ=0.278, q=0.755 | ρ=-0.333, q=0.755 | ρ=-0.042, q=0.934 |
| Right Caudate | ρ=-0.342, q=0.755 | ρ=-0.070, q=0.932 | ρ=0.270, q=0.755 | ρ=0.026, q=0.955 | ρ=-0.058, q=0.934 | ρ=0.031, q=0.951 | ρ=-0.092, q=0.911 | ρ=-0.278, q=0.755 | ρ=0.228, q=0.814 |
| Left Caudate | ρ=-0.324, q=0.755 | ρ=-0.012, q=0.974 | ρ=0.289, q=0.755 | ρ=0.089, q=0.911 | ρ=-0.044, q=0.934 | ρ=-0.002, q=0.993 | ρ=-0.119, q=0.893 | ρ=-0.304, q=0.755 | ρ=0.174, q=0.842 |
| Right Hippocampus | ρ=-0.315, q=0.755 | ρ=-0.223, q=0.814 | ρ=0.208, q=0.820 | ρ=0.165, q=0.842 | ρ=-0.475, q=0.520 | ρ=-0.140, q=0.873 | ρ=-0.242, q=0.814 | ρ=-0.064, q=0.934 | ρ=-0.077, q=0.930 |
| Left Hippocampus | ρ=-0.334, q=0.755 | ρ=-0.250, q=0.798 | ρ=0.205, q=0.822 | ρ=0.108, q=0.900 | ρ=-0.413, q=0.628 | ρ=-0.172, q=0.842 | ρ=-0.159, q=0.842 | ρ=-0.124, q=0.889 | ρ=-0.007, q=0.986 |
| Right Inferior Lateral Ventricle | ρ=-0.185, q=0.842 | ρ=0.235, q=0.814 | ρ=0.067, q=0.932 | ρ=0.080, q=0.930 | ρ=0.272, q=0.755 | ρ=-0.048, q=0.934 | ρ=-0.098, q=0.908 | ρ=-0.138, q=0.875 | ρ=-0.184, q=0.842 |
| Left Inferior Lateral Ventricle | ρ=-0.199, q=0.838 | ρ=0.334, q=0.755 | ρ=0.295, q=0.755 | ρ=0.318, q=0.755 | ρ=0.139, q=0.880 | ρ=-0.014, q=0.974 | ρ=0.078, q=0.930 | ρ=-0.291, q=0.755 | ρ=-0.100, q=0.908 |
| Right Lateral Ventricle | ρ=-0.446, q=0.563 | ρ=0.175, q=0.842 | ρ=0.436, q=0.563 | ρ=0.090, q=0.911 | ρ=0.178, q=0.842 | ρ=-0.361, q=0.755 | ρ=-0.194, q=0.838 | ρ=-0.560, q=0.425 | ρ=-0.175, q=0.842 |
| Left Lateral Ventricle | ρ=-0.372, q=0.755 | ρ=0.231, q=0.814 | ρ=0.443, q=0.563 | ρ=0.187, q=0.842 | ρ=0.120, q=0.893 | ρ=-0.324, q=0.755 | ρ=-0.173, q=0.842 | ρ=-0.569, q=0.425 | ρ=-0.164, q=0.842 |
| Right Pallidum | ρ=-0.445, q=0.563 | ρ=-0.210, q=0.820 | ρ=-0.159, q=0.842 | ρ=-0.196, q=0.838 | ρ=-0.105, q=0.900 | ρ=-0.158, q=0.842 | ρ=-0.012, q=0.974 | ρ=-0.478, q=0.520 | ρ=-0.067, q=0.932 |
| Left Pallidum | ρ=-0.460, q=0.563 | ρ=-0.107, q=0.900 | ρ=0.069, q=0.932 | ρ=-0.149, q=0.861 | ρ=0.028, q=0.955 | ρ=-0.142, q=0.872 | ρ=0.045, q=0.934 | ρ=-0.547, q=0.425 | ρ=-0.058, q=0.934 |
| Right Putamen | ρ=-0.288, q=0.755 | ρ=-0.127, q=0.889 | ρ=0.103, q=0.900 | ρ=-0.157, q=0.842 | ρ=-0.047, q=0.934 | ρ=0.041, q=0.934 | ρ=-0.039, q=0.934 | ρ=-0.300, q=0.755 | ρ=0.230, q=0.814 |
| Left Putamen | ρ=-0.215, q=0.820 | ρ=-0.129, q=0.884 | ρ=0.193, q=0.838 | ρ=-0.072, q=0.932 | ρ=-0.105, q=0.900 | ρ=0.058, q=0.934 | ρ=-0.125, q=0.889 | ρ=-0.205, q=0.822 | ρ=0.163, q=0.842 |
| Right Thalamus | ρ=0.143, q=0.872 | ρ=-0.340, q=0.755 | ρ=0.246, q=0.810 | ρ=-0.272, q=0.755 | ρ=-0.133, q=0.880 | ρ=-0.041, q=0.934 | ρ=0.215, q=0.820 | ρ=-0.219, q=0.820 | ρ=-0.097, q=0.908 |
| Left Thalamus | ρ=0.369, q=0.755 | ρ=-0.617, q=0.425 | ρ=-0.078, q=0.930 | ρ=-0.228, q=0.814 | ρ=-0.580, q=0.425 | ρ=0.262, q=0.785 | ρ=0.167, q=0.842 | ρ=0.142, q=0.872 | ρ=0.278, q=0.755 |
| Right Ventral Diencephalon | ρ=-0.232, q=0.814 | ρ=-0.255, q=0.796 | ρ=0.113, q=0.893 | ρ=-0.412, q=0.628 | ρ=0.059, q=0.934 | ρ=-0.103, q=0.900 | ρ=0.166, q=0.842 | ρ=-0.260, q=0.785 | ρ=-0.045, q=0.934 |
| Left Ventral Diencephalon | ρ=-0.149, q=0.861 | ρ=-0.303, q=0.755 | ρ=0.133, q=0.880 | ρ=-0.360, q=0.755 | ρ=-0.114, q=0.893 | ρ=0.017, q=0.974 | ρ=0.251, q=0.798 | ρ=-0.224, q=0.814 | ρ=0.050, q=0.934 |

*FDR corrected p-values <0.05. T-PANSS: Positive and Negative Symptom Scale total score, P-PANSS: Positive and Negative Symptom Scale positive subscale score, N-PANSS: Positive and Negative Symptom Scale negative subscale score, G-PANSS: Positive and Negative Symptom Scale general subscale score.

Supplementary Table 9. Participants’ demographics and clinical characteristics. (Only the monotherapy patients and healthy controls)

| Variables | TRS (n=18)  Mean (SD) or n | FLR (n=15)  Mean (SD) or n | HC (n=30)  Mean (SD) or n | F/ χ2, p value |
| --- | --- | --- | --- | --- |
| Age (years) | 36.4 (11.70) | 37.2 (8.6) | 39.3 (8.96) | F(2,60) = 0.536,  p = 0.587 |
| Sex (male/female) | 8/10 | 10/5 | 17/13 | χ2(2) = 1.66,  p = 0.435 |
| Education (years) | 12.6 (3.44) | 13.6 (2.92) | 12.7 (3.07) | χ2(2) = 1.65,  p = 0.437 |
| Age of Onset (years) | 22.2 (5.22) | 25.2 (8.77) | - | F(2,31) = 2.230,  p = 0.145 |
| Duration of Illness (years) | 14.5 (9.49) | 14.8 (10.14) | - | F(2,31) = 1.207,  p = 0.280 |
| CPZ antipsychotic dose equivalents (mg/day) | 548.7 (439.99) | 582.4 (387.39) | - | F(2,31) = 0,003,  p = 0.951 |
| PANSS total | 43.9 (9.66) | 46.0 (8.29) | - | F(2,31) = 0.201,  p = 0.657 |
| PANSS positive | 8.1 (1.51) | 8.83 (2.22) | - | F(2,31) = 0.917,  p = 0.346 |
| PANSS negative | 11.7 (3.97) | 13.6 (3.63) | - | F(2,31) = 0.206,  p = 0.653 |
| PANSS general | 23.1 (6.66) | 23.9 (4.65) | - | F(2,31) = 0.133,  p = 0.718 |

TRS: Treatment resistant schizophrenia, FLR: First-line treatment responders, HC: Healthy Controls. PANSS: Positive and Negative Symptom Scale, CPZ: Chlorpromazine.

Supplementary Table 10. Volume differences (mm^3^) in subcortical structures between treatment-resistant, treatment-responsive, and healthy control groups, controlling for age, sex, and total intracranial volume. (Only the monotherapy patients and healthy controls)

| Region of Interest | TRS (n=18)  Mean (SE) | FLR (n=15)  Mean (SE) | HC (n=30)  Mean (SE) | F, p-value | FDR corrected p-value |
| --- | --- | --- | --- | --- | --- |
| Right Accumbens | 343.4 (10.16) | 362.2 (9.72) | 373.6 (6.10) | F (2,57) = 4.905,  p = 0.011^a^ | p = 0.036 |
| Left Accumbens | 380.8 (9.93) | 401.1 (9.93) | 419.2 (6.27) | F (2,57) = 8.018  p = 0.001^b^ | p = 0.004 |
| Right Amygdala | 836.1 (20.12) | 845.1 (19.25) | 935.5 (13.39) | F (2,57) = 16.160,  p < 0.001^c^ | p = 0.004 |
| Left Amygdala | 846.5 (21.44) | 861.8 (20.51) | 947.8 (14.50) | F (2,57) = 13.292,  p < 0.001^d^ | p = 0.004 |
| Right Caudate | 2761.6 (85.43) | 2939.2 (81.72) | 2975.8 (56.94) | F (2,57) = 3.091,  p = 0.053 | p = 0.100 |
| Left Caudate | 2695.9 (79.63) | 2809.1 (76.17) | 2914.7 (54.83) | F (2,57) = 3.845,  p = 0.027 | p = 0.069 |
| Right Hippocampus | 3222.6 (63.50) | 3265.2 (60.75) | 3545.2 (46.59) | F (2,57) = 15.930,  p < 0.001^e^ | p = 0.004 |
| Left Hippocampus | 2998.9 (62.98) | 3123.5 (60.24) | 3302.2 (44.54) | F (2,57) = 12.193,  p < 0.001^f^ | p = 0.004 |
| Right Pallidum | 249.9 (26.99) | 252.7 (25.82) | 200.7 (17.20) | F (2,57) = 2.310,  p = 0.108 | p = 0.155 |
| Left Pallidum | 255.3 (27.89) | 265.7 (26.68) | 202.6 (18.30) | F (2,57) = 2.930,  p = 0.061 | p = 0.100 |
| Right Putamen | 3223.7 (130.25) | 3679.2 (124.60) | 3521.9 (81.24) | F (2,57) = 3.749,  p = 0.030 | p = 0.069 |
| Left Putamen | 3299.4 (135.8) | 3761.2 (129.92) | 3587.6 (87.34) | F (2,57) = 3.607,  p = 0.033 | p = 0.069 |
| Right Thalamus | 4356.7 (120.41) | 4497.7 (157.49) | 4826.7 (10.101) | F (2,57) = 4.455,  p = 0.016^g^ | p = 0.046 |
| Left Thalamus | 3723.1 (158.04) | 4121.3 (151.18) | 4361.3 (97.48) | F (2,57) = 7.193,  p = 0.002^h^ | p = 0.007 |
| Right Ventral Diencephalon | 983.9 (43.03) | 1050.8 (41.17) | 1077.9 (27.80) | F (2,57) = 2.202,  p = 0.120 | p = 0.162 |
| Left Ventral Diencephalon | 1005.7 (43.32) | 1059.7 (41.44) | 1071.7 (27.66) | F (2,57) = 1.032,  p = 0.363 | p = 0.417 |
| Brain Stem | 1380.1 (161.04) | 1408.7 (154.06) | 1400.0 (115.92) | F (2,57) = 0.046,  p = 0.856 | p = 0.856 |
| Third Ventricle | 811.5 (46.13) | 655.5 (51.31) | 632.2 (38.25) | F (2,58) = 2.975,  p = 0.059* | p = 0.100 |
| Fourth Ventricle | 1317.9 (114.44) | 1175.9 (125.98) | 1323.4 (85.04) | F (2,58) = 0.339,  p = 0.713* | p = 0.753 |
| Right Inferior Lateral Ventricle | 95.5 (7.99) | 75.4 (8.80) | 85.9 (6.92) | F (2,58) = 1.187,  p = 0.312* | p = 0.377 |
| Left Inferior Lateral Ventricle | 41.1 (3.98) | 35.7 (4.36) | 35.5 (3.99) | F (2,58) = 0.329,  p = 0.721* | p = 0.757 |
| Right Lateral Ventricle | 8118.2 (696.48) | 6500.7 (766.70) | 5862.5 (694.50) | F (2,58) = 2.422,  p = 0.098* | p = 0.150 |
| Left Lateral Ventricle | 9240.0 (793.73) | 7167.2 (873.76) | 7111.0 (807.26) | F (2,58) = 1.620,  p = 0.207* | p = 0.264 |
| Total Intracranial Volume | 1,452,859.75 (30,566.40) | 1,411,039.18 (33,849.91) | 1,489,735.25 (22,508.62) | F (2,58) = 0.506,  p = 0.605* | - |

TRS: Treatment resistant schizophrenia, FLR: First-line treatment responders, HC: Healthy Controls.

a: False Discovery Rate (FDR) p-value <0.05: TRS<HC (p_tukey_ = 0.020, cohen’s d = -0.84).

b: FDR p-value <0.05: TRS<HC (p_tukey_ = 0.002, cohen’s d = -1.07).

c: FDR p-value <0.05: TRS<HC (p_tukey_ < 0.001, cohen’s d = -1.31), FLR<HC (p_tukey_ = 0.003, cohen’s d = -1.12).

d: FDR p-value <0.05: TRS<HC (p_tukey_ < 0.001, cohen’s d = -1.17), FLR<HC (p_tukey_ = 0.013, cohen’s d = -0.95).

e: FDR p-value <0.05: TRS<HC (p_tukey_ < 0.001, cohen’s d = -1.31), FLR<HC (p_tukey_ = 0.012, cohen’s d = -0.95).

f: FDR p-value <0.05: TRS<HC (p_tukey_ < 0.001, cohen’s d = -1.23).

g: FDR p-value <0.05: TRS<HC (p_tukey_ = 0.014, cohen’s d = -0.87).

h: FDR p-value <0.05: TRS<HC (p_tukey_ < 0.001, cohen’s d = -1.18).

*Controlled for age and sex.

**
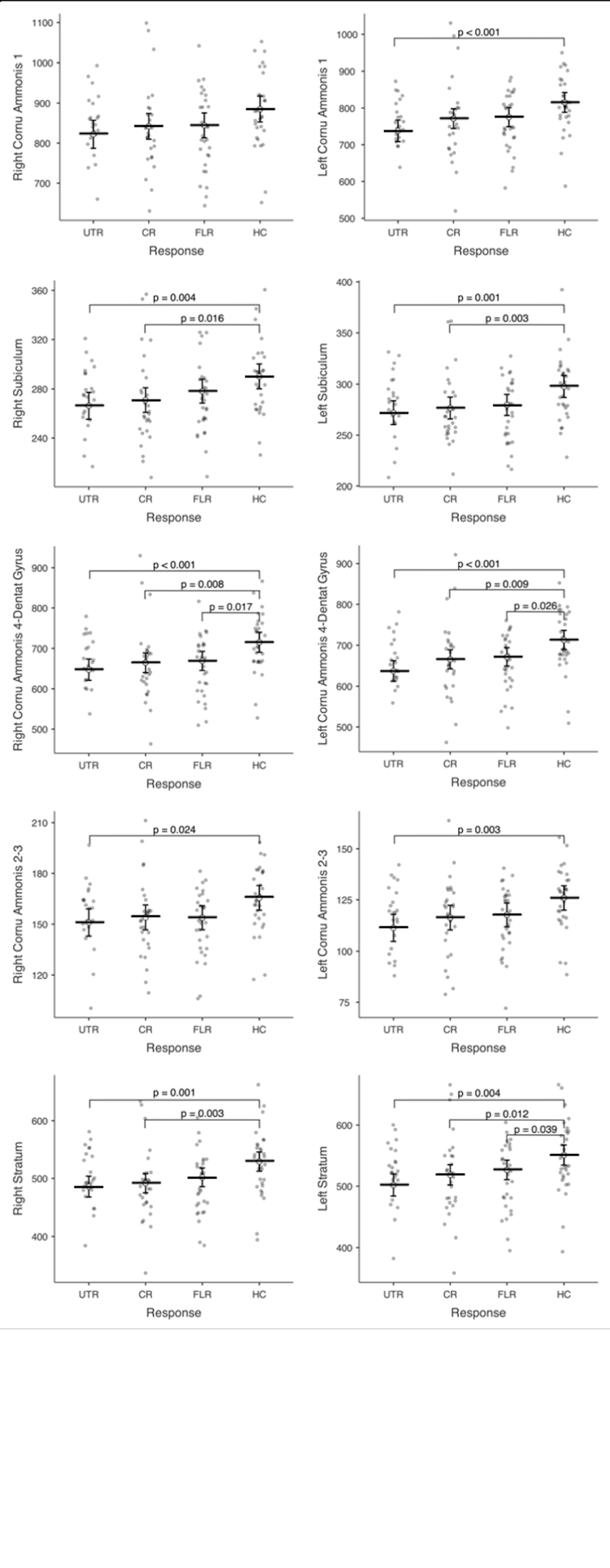
**

**Supplementary Fig 1.** Group comparisons of mean hippocampal subfields (mm³) across four groups: ultra-treatment-resistant (UTR), clozapine-responsive (CR), first-line responders (FLR), and healthy controls (HC). Error bars represent confidence intervals. Statistical significance was assessed using post-hoc Tukey HSD tests, and brackets with corresponding p-values indicate significant between-group differences. All volumetric values were corrected for age, sex, and total intracranial volume (TIV).


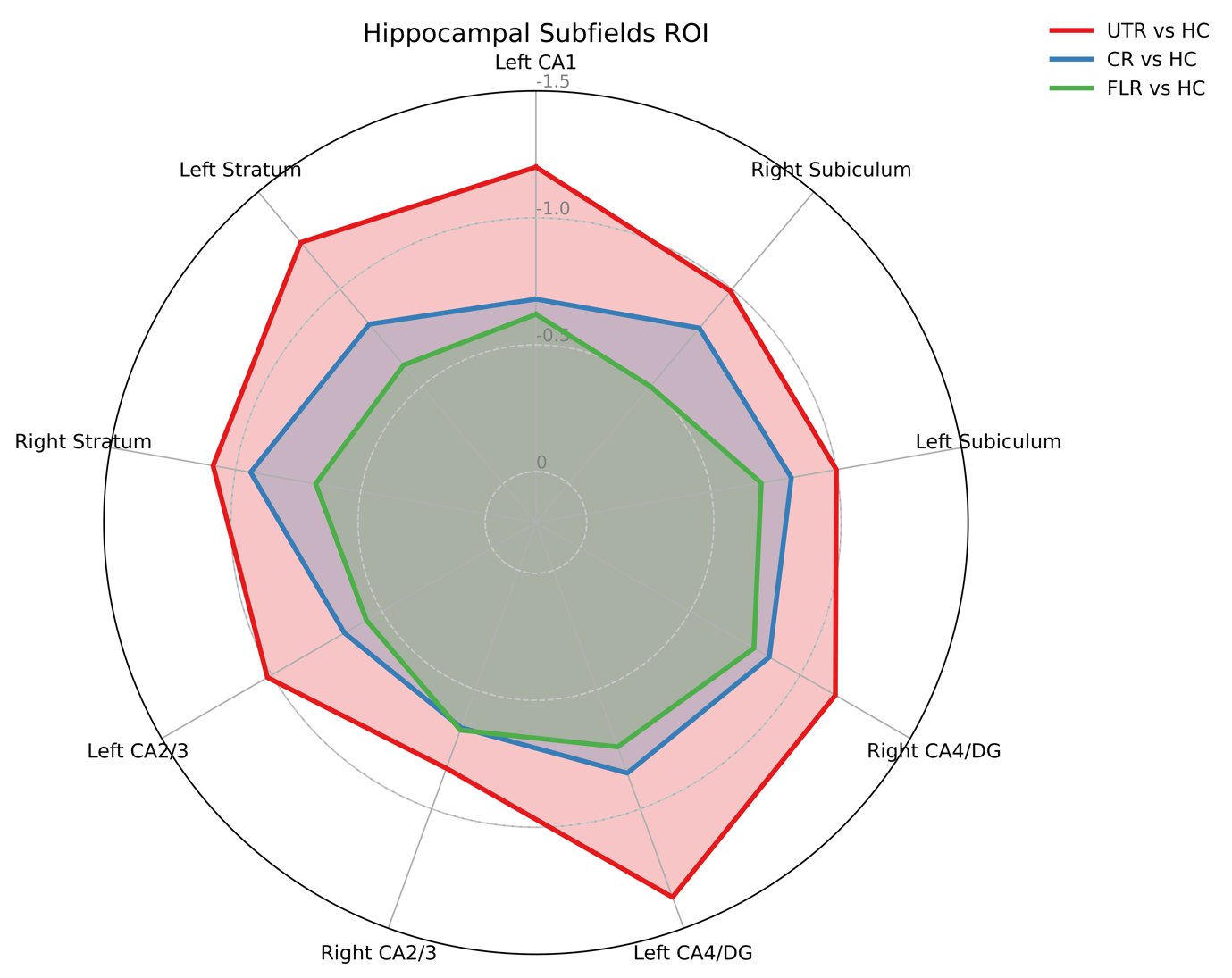


**Supplementary Fig 2.** Radar plot illustrating standardized Cohen’s d effect sizes for each patient subgroup (ultra-treatment-resistant (UTR), clozapine-responsive (CR), and first-line responder (FLR)) relative to healthy controls (HC) across hippocampal subfields.
